# Impact of *Ascophyllum nodosum* extract biostimulants on nutrient use efficiency and seedling establishment in wheat and barley

**DOI:** 10.3389/fpls.2026.1813433

**Published:** 2026-05-12

**Authors:** Elomofe Ikuyinminu, Oscar Goñi, Łukasz Łangowski, Shane O’Connell

**Affiliations:** 1 Brandon Bioscience, Tralee, Ireland; 2 Plant Biostimulant Group, Centre for Applied Bioscience Research, Munster Technological University-Tralee, Tralee, Ireland

**Keywords:** *Ascophyllum nodosum* extract, barley, biomass, biostimulants, nutrient use efficiency, seedling establishment, wheat

## Abstract

**Introduction:**

Seedling emergence and early crop establishment are critical stages for crop productivity, yet they are highly vulnerable to abiotic stress and nutrient limitation. Although plant biostimulants have emerged as promising tools to enhance crop resilience, their influence on micronutrient dynamics during early development remains poorly understood. This study provides one of the first integrated assessments of micronutrient uptake and remobilisation in two major cereal crops, winter barley and wheat, during early seedling growth.

**Methods:**

Three commercial Ascophyllum nodosum extracts (ANE A, ANE B and ANE C) were compared to determine whether compositional differences translated into distinct physiological responses in wheat. Under controlled in−vitro conditions using nutrient−free (0 MS) and reduced−nutrient (1/10 MS) media, ANE A was applied either foliarly or directly to the growth medium. Its influence on nutrients uptake and remobilisation was assessed using ICP-MS.

**Results:**

The extracts showed clear functional divergence: ANE A and ANE C significantly increased biomass, with ANE A primarily enhancing root growth and uniquely increasing several micronutrients, while all extracts improved selected macro− and secondary nutrients. Based on its performance, ANE A was selected for detailed mechanistic evaluation. Across both cereals, ANE A significantly increased root biomass (12–26% over controls) and induced distinct shifts in macro− (K, P), secondary− (Ca, Mg) and micronutrient (B, Mn, Cu, Fe, Mo, Zn) accumulation and partitioning. Nutrient use efficiency metrics further confirmed enhanced uptake and translocation, indicating an active role of ANE A in micronutrient mobilisation.

**Discussion:**

By revealing how ANE A reprograms early nutrient allocation and improves micronutrient dynamics under nutrient-limited conditions, this study advances mechanistic understanding of ANE−based biostimulants and supports their potential use in resilient, sustainable nutrient management strategies for cereal production.

## 1 Introduction

The early stages of crop growth, encompassing seed germination, seedling emergence, and initial establishment, significantly influence the final agricultural productivity (Atar et al., 2020). However, the efficient and uniform establishment of plants in the field is challenged by various environmental stresses and nutrient deficiencies (Reed et al., 2022; Welch, 2024). Early nutrient deficiency renders crops highly susceptible to environmental stresses (Cakmak, 2005; Hajiboland, 2012; Nieves-Cordones et al., 2019; Pandey et al., 2020), underscoring the need for adequate nutritional status in seeds and seedlings to support robust early growth. Of the 16 essential elements required for the proper plant growth and development, 13 are derived solely from the soil or growth medium if no fertilizer is applied (Uchida, 2000). Most of the nutrients absorbed by the plants correspond to the macronutrients nitrogen (N), phosphorus (P) and potassium (K), which play multiple roles at structural and physiological level (Pandey et al., 2020; Toor et al., 2021; Kumari et al., 2022). Secondary nutrients such as calcium (Ca) and magnesium (Mg) are involved in root development, cell structure, photosynthesis, and nutrient uptake processes in early growth stages (White and Broadley, 2003; Mengutay et al., 2013; Kumari et al., 2022). Micronutrients such as boron (B), zinc (Zn), iron (Fe), manganese (Mn), copper (Cu) and molybdenum (Mo) are required in small amounts, but their presence in plant tissues is critical for an efficient plant metabolism, regulation of nutrients, chlorophyll biosynthesis, generation of carbohydrates, reproductive growth, and development of fruit and seed (Tripathi et al., 2015; Rahman et al., 2020).

Wheat (*Triticum* spp.) and barley (*Hordeum vulgare L.*) are two cereal crops that belong to the family Poaceae (order Poales). They are cornerstone crops in global agriculture, vital for food security, economic stability, and future-proofing agriculture. While wheat is the world’s top cereal in both production and trade, barley is the fourth-most-produced cereal, essential for feed, brewing, and it is also considered a principal food in regions where other major cereals cannot be grown (Giraldo et al., 2019). Wheat and barley-based cropping systems are sensitive to abiotic stress conditions and nutrient deficiencies during the sowing and early establishment period. Such deficiencies can cause significant alteration in physiological and biochemical processes during seedling establishment, leading to adverse consequences on crop growth and productivity (Smith et al., 1999; Mäkinen et al., 2018; Pandey et al., 2020; Heil et al., 2021; Vuković et al., 2022).

Different approaches have been suggested to overcome cereal production limitations because of poor stand establishment and nutrient deficiencies. The viability and vigour of seeds can be improved by techniques generally known as seed priming, which enhance the speed and uniformity of germination, seedling growth and final yield of barley and wheat crops. However, factors such as the nature of the soaking solution, temperature, oxygen supply in the soil, and storage conditions affect the efficiency of the treatment and the longevity of the primed seed (Ajouri et al., 2004; Abdulrahmani et al., 2007; Salehzade et al., 2009; Yari et al., 2011; Farooq et al., 2012; Khafagy et al., 2017; Farooq et al., 2019). The screening of cereal genotypes for desired traits is the most common approach. However, in wheat breeding, the efforts to improve early vigour through a more efficient uptake and use of nutrient resources in the early growing season have mainly focused on N (Cormier et al., 2016; López-Arredondo et al., 2017). Because plant growth in these stages can be co-limited by more than a single nutrient, it would be important to explore their relevance and relationship with a more efficient early crop establishment (Weih et al., 2018; Liu et al., 2021). Targeted fertilisation and novel delivery systems such as nanofertilizers have been proposed to optimize NUE and reduce environmental impact (Saquee et al., 2023; Semenova et al., 2024; Khundi et al., 2025). However, such approaches often neglect the early developmental window, where nutrient remobilisation from seed reserves plays a crucial compensatory role under nutrient deprivation.

Plant biostimulants have emerged as an integral component of modern and sustainable agriculture to enhance crop productivity under mounting environmental challenges. These naturally derived substances including seaweed extracts, humic extracts, protein hydrolysates, and beneficial microorganisms, stimulate plant physiological processes to improve nutrient use efficiency and abiotic stress tolerance leading to a higher crop yield and quality (Rouphael and Colla, 2020). Extensive published research supports the use of seaweed extracts to enhance nutrient uptake and seedling establishment in a sustainable manner (Rayorath et al., 2008; Sangha et al., 2014; Ali et al., 2021). Specifically, *Ascophyllum nodosum* extract (ANE) biostimulants can improve uptake and transport of macro-, secondary and micronutrients in *Arabidopsis thaliana* and crops such as avocado, barley, durum wheat, grapevine, maize, oilseed rape, tomato, snap bean and winter wheat (Turan and Köse, 2004; Abou El-Yazied et al., 2012; Jannin et al., 2013; Billard et al., 2014; Sabir, 2014; Stamatiadis et al., 2015; Ali et al., 2016; Goñi et al., 2016; Ertani et al., 2018; Laurent et al., 2020; Goñi et al., 2021; Shukla and Prithiviraj, 2021; Łangowski et al., 2022; Quille et al., 2025). However, despite extensive documentation of ANE effects on mature plant physiology, their role in driving early root-shoot development and micronutrient remobilisation from seed reserves remains poorly understood. Despite progress in understanding how specific ANE biostimulants can improve N uptake and assimilation pathways (Laurent et al., 2020; Goñi et al., 2021; Łangowski et al., 2022), no comprehensive studies to date have quantified how ANE treatments influence the coordinated uptake and internal translocation of both macro- and micronutrients during the early seedling phase, an essential yet transient stage of crop establishment.

After compositional characterisation of three commercial *Ascophyllum nodosum* extracts (ANE A, ANE B and ANE C), a comparative screening in wheat seedlings was performed to establish whether differences in extraction processes and composition translated into distinct biostimulant responses. This initial assessment enabled the identification of ANE A as the most effective extract based on its capacity to improve biomass accumulation and nutrient content. Building on these findings, the present study provides a detailed evaluation of how ANE A influences macro−, secondary− and micronutrient uptake and remobilisation in barley and wheat seedlings during early development. By applying ANE A either as a foliar treatment or directly to the root system under nutrient−free (0 MS) and reduced−nutrient (1/10 MS) conditions, its effects on biomass, tissue−specific nutrient allocation, and nutrient use efficiency (NUE) markers for each nutrient such as; apparent recovery efficiency (RE) and agronomic efficiency (AE) indices were quantified. The *in-vitro* agar system not only excludes microbial influences but also ensures consistent, controlled nutrient availability, facilitating precise evaluation of biostimulant effects. 1/10 MS agar medium was considered nutrient deficient for wheat seedlings based on the reduced biomass accumulation under controlled experimental conditions (Łangowski et al., 2022). This integrated approach provides new mechanistic insight into how ANE-derived biostimulants modulate nutrient dynamics during the most vulnerable stage of cereal establishment and offers a foundation for developing more efficient, sustainable nutrient management strategies.

## 2 Materials and methods

### 2.1 Materials

Three commercially available liquid ANEs were applied to wheat plants as biostimulant treatments (ANE A, ANE B and ANE C). ANE A, a highly concentrated commercial ANE biostimulant, is produced by Brandon Bioscience using a proprietary extraction at high temperatures and alkaline conditions. ANE B and ANE C are manufactured using other proprietary extraction at high temperatures and alkaline conditions. All chemical reagents and standards used for the experiments were purchased from Sigma-Aldrich (Arklow, Ireland) and Agilent Technologies (Cork, Ireland).

### 2.2 Chemical characterization of utilised biostimulants

Chemical characterisation was performed as outlined in Goñi et al. (2016). The total solids were determined by drying samples in a convection oven at 105 °C for 18 h. The same dried samples were subsequently used to determine ash content by incineration in a furnace at 550 °C for 6 h. Uronic acids content was determined using metahydroxydiphenyl method, with alginic acid used as a calibration standard. Samples were digested in 0.0125 M sodium tetraborate in sulfuric acid at 100 °C, and absorbance was measured at 520 nm. Fucoidan was determined according to the cysteine-sulfuric acid method using L-fucose as a standard. Samples were digested in a sulfuric acid/water mixture (6:1) at 100 °C and then allowed to cool in an ice bath before the colour-forming agent (3% w/v L-cysteine in HCl) was added. Colour was allowed to form at room temperature for an hour before absorbance was measured at 400 nm and corrected at 460 nm. Laminarin and free mannitol determination was performed (at room temperature) by HPAEC-PAD using a Carbopac PA-100 anion-exchange resin (4.6 × 250 mm) connected to a Carbopac PA-100 guard column (4.6 × 50 mm) (Thermo Scientific Dionex, Ireland). The total polyphenol content was measured using Folin− Ciocalteu’s phenol reagent, and phloroglucinol was used as the standard. The content of other organic components was calculated by differences to the total organic amount.

### 2.3 Plant material, growth conditions and ANE treatment application

Wheat seedings (cv. Graham) were grown according to Łangowski et al., 2022. For primary screening and consistent treatment application, ANE A, ANE B and ANE C were dried to powder at 90 °C and mixed homogenously with liquid medium before agar gelling. All ANE treatments were standardized based on total solids content (5 mg per treatment) to enable comparison of intrinsic biological activity across extracts with differing formulation concentrations. The addition of the same dry amount of ANE was equivalent to a field application of 2 L/Ha (ANE A and ANE B) and 4 L/Ha (ANE C), assuming an application tank of 300 L/Ha.

Barley (cv. Belfry) and wheat (cv. Graham) seedlings were grown in a growth chamber under controlled conditions (19/14 °C with 16 h of light and 8 h of darkness and 80 ± 5% RH under a light intensity of 120 μmol m−2·s−1) for 6 days. Initially, barley and wheat seeds were sterilised with 0.5% v/v sodium hypochlorite for 5 min and subsequently rinsed multiple times with sterile water. Barley and wheat seeds were then sown on 2% w/v agar medium without any supplementation for 6 days. Subsequently, barley and wheat seedlings of similar size at BBCH 10-11 (first leaf through coleoptile to 1^st^ leaf unfolded), were transferred to 500ml containers filled with 75 ml of 2% agar w/v growth medium without any nutrient supplementation (0 MS) while others were transferred to 500 ml containers filled with 75ml of 2% agar w/v growth medium supplemented with a reduced nutrient rate of 1/10 MS (Murashige – Skoog). Containers with growth medium were placed randomly in the growth chamber and their position was changed every 2 days to avoid any positional effect. The concentration of the nutrients in this growth media were as follows: 84.02 mg/L N; 78.38 mg/L K; 0.23 mg/L Na;1.12 mg/L Fe; 11.99 mg/L Ca; 1.78 mg/L Mg; 3.87 mg/L P; 0.11 mg/L B; 0.00062 mg/L Co; 0.55 mg/L Mn; 0.20 mg/L Zn; 0.00064 mg/L Cu; 0.0099 mg/L Mo. In comparison, tested biostimulants contain insignificant nutrients content, too low to impact NUE. For convenience and consistent treatment application in subsequent experiments, ANE A was dried to powder at 90 °C and either mixed homogenously with liquid medium before agar gelling (direct application of 5 mg to growth medium to the root system) or dissolved with water (foliar application of 1 mL of product at 5 mg/mL). The amount of biostimulant applied in both treatments was equivalent to a rate of 2 L/Ha for a cereal crop grown under field conditions assuming an application tank of 300 L/Ha. For the control treatments in the foliar application groups, 1 mL sterile water was applied to the seedlings, not adding anything to the agar medium. Seedlings were grown for 6 days under the light and temperature conditions described above until BBCH 12-13 (2^nd^ to 3^rd^ leaf unfolded). At the end of the experiment, 12 day-plant seedlings were harvested, root and shoot tissue separated with a blade. Root and shoot biomass were determined and expressed as fresh weight (FW). Seedling samples were dried at 100 °C for 24 h to determine their moisture content, dry weight (DW) and nutrient quantification analysis.

### 2.4 Nutrient quantification in root and shoot tissues

Root and shoot samples (50–100 mg) were dissolved with 2 mL of high purity Suprapur^®^ 65% nitric acid (HNO_3_) and digested using the CEM MARS 6 - Microwave Digestion System (CEM Corporation, Matthews, NC, USA). Quantification of total K, P, Ca, Mg, B, Mn, Cu, Fe, Mo and Zn was performed using an inductively coupled plasma mass spectrometer (Agilent ICP MS 7800, Agilent Technologies, Santa Clara, CA, USA). Nutrients were identified by comparison to commercial standards, quantified by peak integration and concentrations expressed as % w/w DW tissue. Nutrient content in each tissue was later calculated using the biomass results obtained at the end of the experiment and their moisture content and expressed as total mg, µg or ng for each tissue.

### 2.5 Calculation of NUE parameters

RE and AE calculations were adapted from Dobermann (2007) and applied to each nutrient and tissue described above for both wheat and barley seedlings.

RE = Apparent crop recovery efficiency of supplied nutrient
*RE = (U – U_0_)/F*
AE = Agronomic efficiency of supplied nutrients
*AE = (Y -Y_0_)/F*

Where:

F: amount of each nutrient made available to the seedlings in the 1/10 MS growth medium
U: content of nutrient acquired by the root or shoot in 1/10 MS
U_0_: content of nutrient acquired by the root or shoot in 0 MS
Y: Root or shoot biomass in 1/10 MS.
Y_0_: Root or shoot biomass in 0 MS.

RE and AE values were expressed as mg nutrient tissue/g supplied nutrient and g biomass/g supplied nutrient, respectively, and expressed as percentage change with respect to the control.

### 2.6 Statistical analysis

Phenotypic assessment of the winter barley and wheat grown in plastic containers was performed using at least 15 independent biological replicates, with 7 plants per replicate. Nutrient quantification was performed in at least four biological replicates for each treatment, condition and tissue using the plant samples described above. Unless stated otherwise, all data are expressed as mean ± standard error (SE). Statistics were evaluated with Sigma Plot 12 and Statgraphics Centurion XVI software. Principal component analysis (PCA) was conducted to determine correlations among the various compositional markers, phenotypic traits, and nutrient contents. The PCA was performed using the XLSTAT software package, version 2014.5.03, based on the Pearson correlation matrix (n-1). The correlation biplot was generated using the first and second principal components (PCs). The effect of nutrient condition and treatment on phenotypic and nutrient quantification data was determined using the two-way ANOVA with Tukey’s HSD test (p ≤ 0.05). Where the interaction (N × B) between the two factors condition (N) and treatment (B) was significant, data were subjected to either t-test or one-way ANOVA by Tukey’s HSD test at p ≤ 0.05, comparing the treatment versus the control within the same growth condition (0 MS vs 1/10 MS). The effect of condition and treatment was evaluated separately as well, comparing the respective means through either a t-test at p ≤ 0.05 or one-way ANOVA by Tukey’s HSD test at p ≤ 0.05. The effect of treatment on NUE parameters for each tissue and crop was evaluated through t-test at p ≤ 0.05. The application of all parametric tests was performed after checking the data normality (Shapiro–Wilk test) and equal variance assumptions. Details of the individual sample size for each analysis and statistical test used are mentioned in the tables and figure legends.

## 3 Results

### 3.1 Compositional analysis of ANE biostimulants

The results presented in **Table 1** provide a compositional evaluation of the three ANE biostimulants, quantifying key components such as ash and major organic fractions. All measurements are expressed relative to the volume of the liquid extract (w/v) rather than on a dry-weight basis. Each biostimulant contained characteristic seaweed derived carbohydrate such as alginate, fucoidan and laminarin (reported as uronic acids, fucose and glucose), as well as free mannitol and polyphenols. Statistically significant differences were observed among the extracts when expressed on this volume basis. ANE A showed higher concentrations of +59% and +105% uronic acids and +38% and +3.3-fold fucose compared with ANE B and ANE C, respectively. ANE B was more enriched in total solids and other organic matter than the other two extracts. In contrast, both ANE A and ANE B contained higher levels of (+2.1-fold, +2.1-fold) ash, (+5-fold, +3.7-fold) laminarin, (+3.1-fold, +2.7-fold) free mannitol and (+2.8-fold, +3.1-fold) polyphenols respectively than ANE C. Overall, ANE C consistently exhibited the lowest values across all measured parameters.

**Table 1:** Compositional analysis of three ANE biostimulant treatments.

Component % (w/v)	ANE biostimulant treatment		
	ANE A	ANE B	ANE C
Total solids	48.0 ± 0.8 b	51.0 ± 0.9 c	22.9 ± 0.7 a
Ash	20.9 ± 0.4 b	21.0 ± 1.0 b	10.0 ± 0.3 a
Uronic acids	4.3 ± 0.5 b	2.7 ± 0.3 a	2.1 ± 0.3 a
Fucose	4.0 ± 0.4 c	2.9 ± 0.2 b	1.2 ± 0.2 a
Laminarin	1.5 ± 0.2 b	1.1 ± 0.2 b	0.3 ± 0.1 a
Free mannitol	2.8 ± 0.3 b	2.4 ± 0.2 b	0.9 ± 0.1 a
Polyphenols	6.1 ± 0.4 b	6.9 ± 0.5 b	2.2 ± 0.2 a
Other organic content	8.4 ± 0.4 b	14.0 ± 0.6 c	6.2 ± 0.3 a

Different letters within the same parameter indicate statistical differences with *p* ≤ 0.05 based on one way ANOVA. Number of biological replicates (n ≥ 3).

### 3.2 Effect of ANE biostimulants on phenotypic markers and nutrient concentrations of wheat

**Figure 1:**
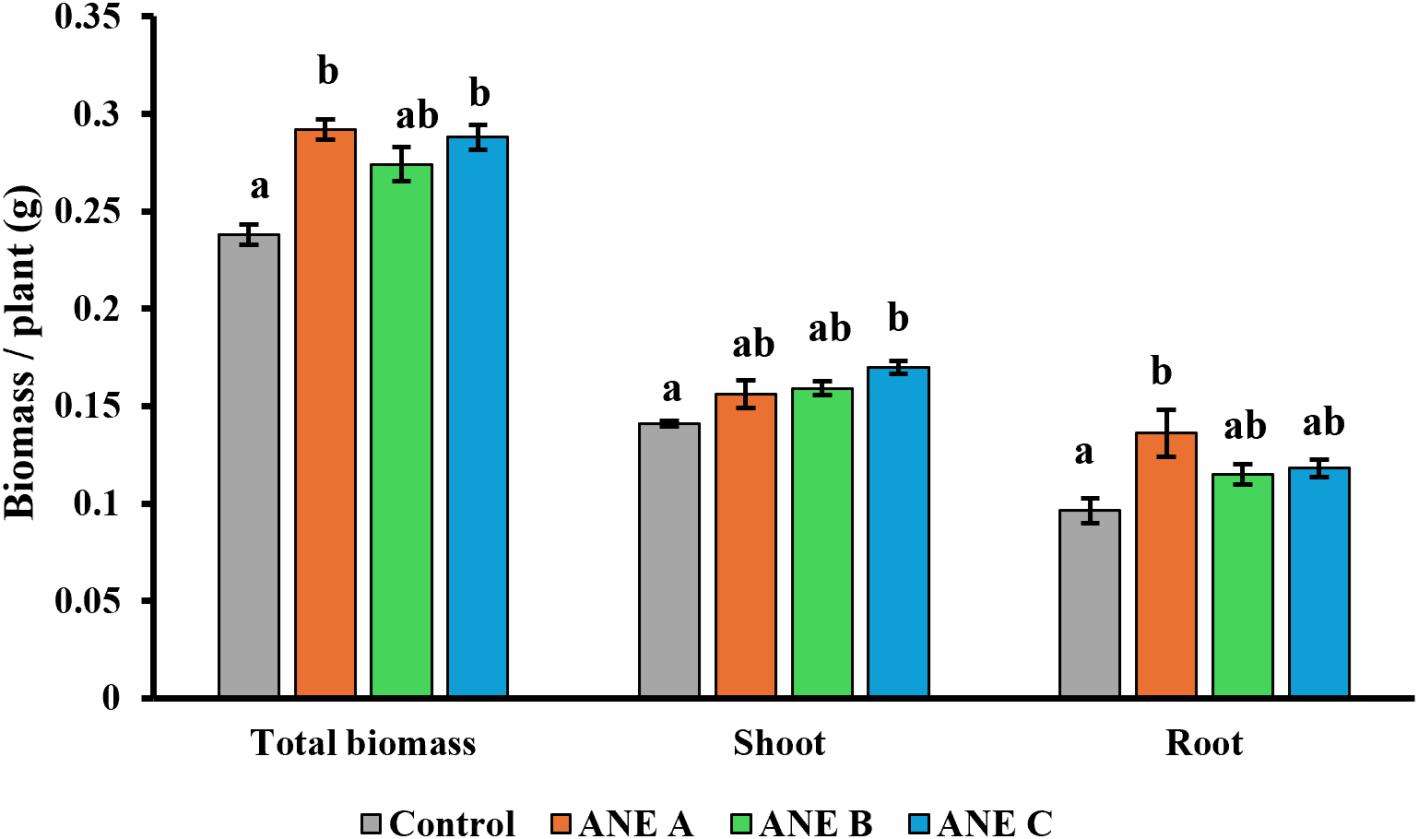
Effect of ANE biostimulants on wheat phenotypic markers. All data are expressed as average per sample in 12-day old plant seedlings. Different letters indicate statistical differences with *p* ≤ 0.05 based on one way ANOVA. Number of biological replicates (n ≥ 3).

The selected ANE biostimulants showed variation in total solids content (%w/v), most likely due to differences in their extraction processes. To ensure a fair comparison of biostimulant activity, treatments were standardised by applying the same amount of total solids, as detailed in the Materials and Methods section. However, because the total solids content differs among the products, this standardisation corresponds to different equivalent agronomic application rates (approximately 2 L/ha for ANE A and ANE B, and 4 L/ha for ANE C). Total shoot and root biomass of winter wheat seedlings were measured six days after treatment. Both ANE A and ANE C significantly increased total biomass by +22.7% and +21%, respectively, compared with the untreated control (**Figure 1**). ANE C primarily stimulated shoot biomass (+20.6%), whereas ANE A produced a strong increase in root biomass (+41.1%). In contrast, ANE B did not produce any statistically significant effect on the measured parameters.

When nutrient contents in wheat seedlings were evaluated relative to the untreated control, ANE A induced significant increases of K + 41%, Ca +70%, P + 31%, Mg +51%, B + 27%, Mn +66%, Mo +96% and Zn +60%. ANE B also enhanced nutrient accumulation, producing significant increases of K + 26%, Ca +50%, P + 16%, Mg +30% and Mn +38%. ANE C resulted in significant increases of K + 29%, Ca +68%, P + 16%, Mg +36%, Mn +72% and Mo +105% (**Table 2**).

**Table 2:** Effect of ANE biostimulants on nutrient contents of wheat seedlings.

Nutrient Content	Control	ANE A	ANE B	ANE C
K (mg)	4.98 a	7.04 b	6.26 b	6.44 b
Ca (mg)	0.15 a	0.25 b	0.22 b	0.24 b
P (mg)	1.22 a	1.60 c	1.41 b	1.41 b
Mg (mg)	0.25 a	0–38 c	0.33 b	0.35 bc
B (µg)	1.39 a	1.76 b	1.50 ab	1.45 a
Mn (µg)	9.88 a	16.40 c	13.60 b	17.00 c
Cu (µg)	1.62 ns	2.88 ns	2.91 ns	2.19 ns
Fe (µg)	26.00 ns	26.50 ns	23.20 ns	25.50 ns
Mo (ng)	81 a	159 b	115 a	166 b
Zn (µg)	8.24 a	13.20 b	9.67 a	10.40 ab

All data are expressed as average per sample in 12-day old plant seedlings. Different letters indicate statistical differences with *p* ≤ 0.05 based on one way ANOVA. Number of biological replicates (n ≥ 3).

### 3.3 PCA evaluation

**Figure 2:**
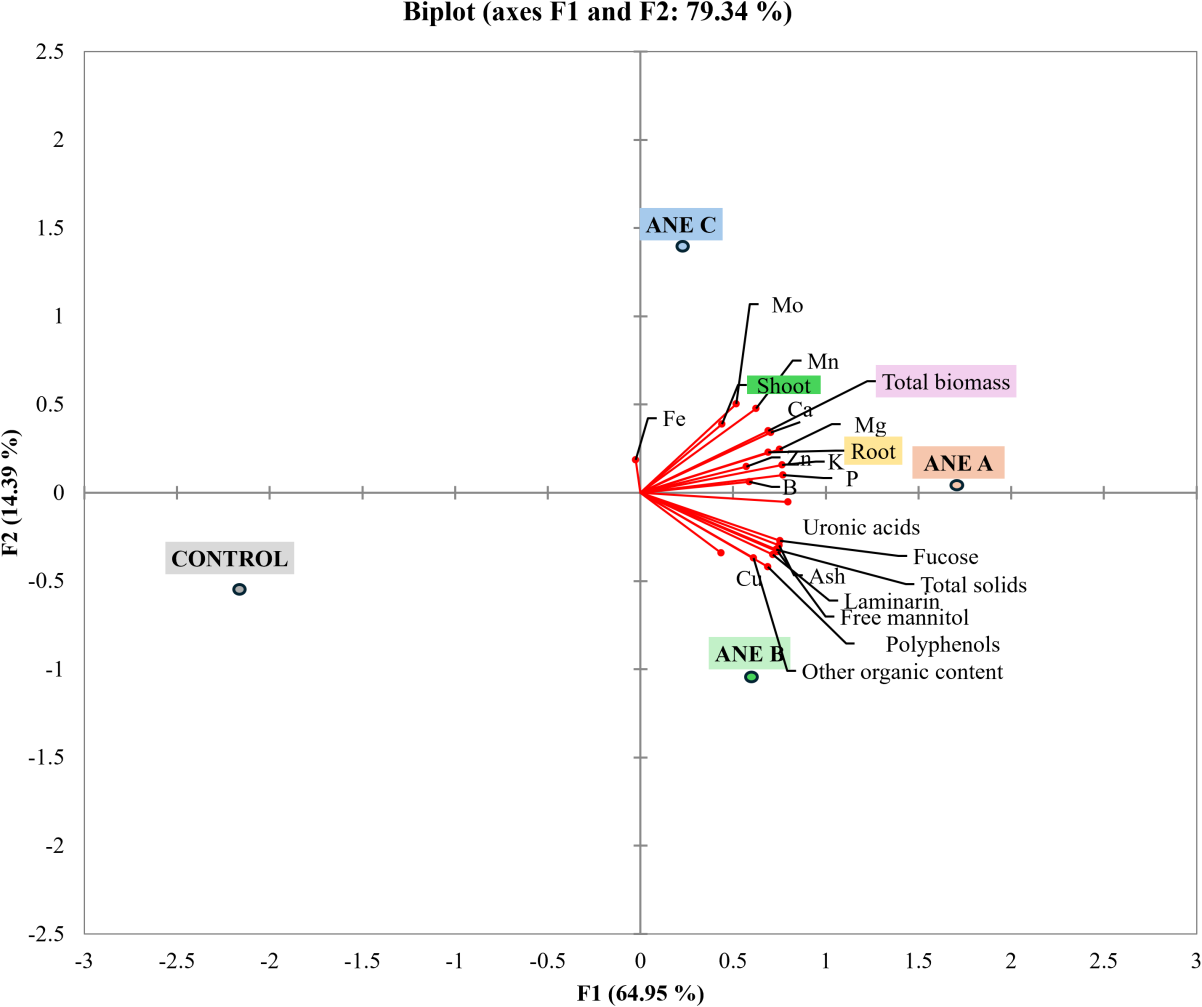
PCA scatter plot based on the first two principal components (PC1 and PC2) generated from the analysed compositional analyses, phenotypical and nutrient contents in wheat seedlings. Observations corresponding to the four samples were included in the PCA scatter plot: control, ANE A, ANE B and ANE C. Number of biological replicates (n ≥ 3).

A PCA model incorporating 21 compositional markers, phenotypic traits, and nutrient contents was developed to provide an integrated overview of how the different ANE treatments influenced biomass production and nutrient status in wheat seedlings (**Figure 2**). The first two principal components explained 79.34% of the total variance, indicating that the biplot offers a robust representation of the dataset. PC1 was strongly and positively associated with nearly all measured variables, except for Fe, which showed a slight negative correlation. PC2 was positively associated with shoot biomass, root biomass, total biomass, and the biomass−adjusted contents of Mo, Mn, Ca, Mg, Zn, K, P and B, while exhibiting negative associations with the remaining variables.

The control group was clearly separated from all ANE treatments, reflecting lower biomass, and nutrient contents. ANE A associated most strongly with biomass and the nutrients K, P, Mg, Zn and B. ANE B aligned more strongly with two ANE compositional markers (other organic and polyphenol content) and the nutrient Cu. ANE C was primarily associated with shoot biomass and the micronutrients Mo and Mn. Other ANE compositional markers such as total solids, fucose, laminarin, free mannitol and uronic acids were aligned between ANE A and ANE B.

Based on the biomass and nutrient content results, ANE A elicited the strongest overall response. Therefore, a more detailed investigation of the effects of ANE A on nutrient uptake and remobilization in barley and wheat seedling was subsequently conducted.

### 3.4 Effect of nutrient conditions and ANE A on phenotypic markers of barley and wheat

This section provides a side-by-side visual and quantitative assessment of both foliar and root application of ANE A biostimulant in barley and wheat seedlings under a dual-nutrient regime (0 MS and 1/10 MS) (**Supplementary Figure 1**; **Tables 3**, **4**). The presence of reduced nutrient conditions (1/10 MS) significantly improved root and shoot biomass in both crops by 29.1% and 58.4% compared to 0 MS conditions, respectively (p ≤ 0.001). The single foliar application of ANE A increased the root biomass of barley and wheat compared to the control under 0 MS and 1/10 MS conditions by 12.2% and 26%, respectively (**Table 3**). Minor increases in shoot biomass were found after foliar application of the biostimulant (3.3% and 5.3% for barley and wheat, respectively) but they were not statistically significant (**Table 3**).

**Table 3:** Effect of nutrient conditions and foliar application of ANE A on phenotypic markers of barley and wheat.

Source of variance	Barley		Wheat	
	Root (g FW)	Shoot (g FW)	Root (g FW)	Shoot (g FW)
Nutrient (N)				
0 MS	0.103 a	0.173 a	0.086 a	0.089 a
1/10 MS	0.141 b	0.249 b	0.111 b	0.141 b
Biostimulant (B)				
Control	0.115 a	0.207	0.087 a	0.112
ANE A	0.129 b	0.214	0.110 b	0.118
N X B				
0 MS x Control	0.099	0.167	0.076	0.086
0 MS x ANE A	0.107	0.179	0.096	0.092
1/10 MS x Control	0.131	0.248	0.098	0.138
1/10 MS x ANE A	0.151	0.250	0.124	0.144
Statistical significance				
Nutrient (N)	***	***	***	***
Biostimulant (B)	**	ns	***	ns
N × B	ns	ns	ns	ns

All data are expressed as average of 12-day old plant seedlings. ns, *, **, and *** means non-significant or significant at *p* ≤ 0.05, *p* ≤ 0.01, and *p* ≤ 0.001, respectively. Different letters indicate statistical differences with *p* ≤ 0.05 based on t-test for N and B factors. Number of biological replicates (n ≥ 15).

**Table 4:** Effect of nutrient conditions and root application of ANE A on phenotypic markers of barley and wheat.

Source of variance	Barley		Wheat	
	Root (g FW)	Shoot (g FW)	Root (g FW)	Shoot (g FW)
Nutrient (N)				
0 MS	0.090 a	0.153 a	0.079 a	0.117 a
1/10 MS	0.104 b	0.199 b	0.106 b	0.155 b
Biostimulant (B)				
Control	0.091 a	0.173	0.084 a	0.132 a
ANE A	0.103 b	0.179	0.101 b	0.140 b
N X B				
0 MS x Control	0.086	0.151	0.071	0.113
0 MS x ANE A	0.094	0.156	0.088	0.122
1/10 MS x Control	0.096	0.195	0.097	0.151
1/10 MS x ANE A	0.111	0.203	0.114	0.159
Statistical significance				
Nutrient (N)	***	***	***	***
Biostimulant (B)	***	ns	***	*
N × B	ns	ns	ns	ns

All data are expressed as average of 12-day old plant seedlings. ns, *, **, and *** means non-significant or significant at *p* ≤ 0.05, *p* ≤ 0.01, and *p* ≤ 0.001, respectively. Different letters indicate statistical differences with *p* ≤ 0.05 based on t-test for N and B factors. Number of biological replicates (n ≥ 15).

A similar trend in the results was observed in the experimental system where the biostimulant was applied directly to the root system. The presence of reduced nutrient conditions (1/10 MS) had an analogous positive effect increasing root and shoot biomass in both crops compared to 0 MS growth medium (p ≤ 0.001). The root application of ANE A increased root biomass of barley and wheat compared to the control under 0 MS and 1/10 MS conditions by 13.3% and 20.1%, respectively. As observed with the foliar application, ANE A was also able to improve shoot biomass in both crops. In wheat the differences in the effects of the biostimulant on this trait (+6.1%) were significant (p ≤ 0.05) (**Table 4**).

To assess the response trends in both application methods, t-tests were done on the relative change of ANE A treated crops with respect to the controls in both wheat and barley (**Table 5**). No statistical significance was observed in the different parameters.

**Table 5:** Effect of ANE A application method on relative changes in phenotypic markers of barley and wheat.

Biostimulant Application Method	Barley		Wheat	
	Boot biomass (%)	Shoot biomass (%)	Root biomass (%)	Shoot biomass (%)
Foliar	+11.69	+3.79	+26.00	+6.46
Root	+14.18	+3.94	+20.63	+7.53
p-value	0.656	0.963	0.415	0.768

All data are expressed as percentage change with respect to the control per root/shoot sample in 12-day old plant seedlings.

### 3.5 Effect of nutrient conditions and ANE A on macro- and secondary nutrients content of barley and wheat

A comprehensive mineral profile of roots and shoots was obtained by combining nutrient concentration, biomass, and moisture data. Reduced nutrient supply (1/10 MS) significantly increased shoot moisture in both crops compared to 0 MS (p ≤ 0.05; **Supplementary Tables 1**, **S2**).

Under 1/10 MS, barley accumulated more K, Ca, P, and Mg than in 0 MS conditions (p ≤ 0.001; **Supplementary Tables 3**, **S4**). The largest differences were for K and Ca (2–4.9-fold higher in both tissues), while P and Mg showed moderate increases (roots: P + 27–68%, Mg −8 to +14%; shoots: P + 41–90%, Mg +28–55%). ANE A enhanced this trend, with both application modes increasing nutrient content but with distinct tissue patterns. Foliar application strongly promoted K accumulation in roots (+45–48%, p ≤ 0.001) and shoots (+25%, p ≤ 0.001), while Ca and P gains were largely restricted to roots (Ca: +6% and +33% in 0 and 1/10 MS, respectively, p ≤ 0.001; P: +8% in both 0 and 1/10 MS, respectively, p ≤ 0.05). Root application increased P in both tissues (+6–8%, p ≤ 0.05), raised Ca in shoots (+11% and +17% in 0 and 1/10 MS, p ≤ 0.001), but reduced it in roots (−40%, p ≤ 0.05). Mg also showed opposite responses depending on the application mode: foliar treatment increased root Mg (+14%, p ≤ 0.05), while root application raised shoot Mg (+9%, p ≤ 0.05).

Wheat seedlings also responded strongly to 1/10 MS, with significant increases in K, Ca, P, and Mg across tissues compared to 0 MS (p ≤ 0.001; **Supplementary Tables 5**, **S6**). K and Ca rose 1.5–4.4-fold, P was more responsive in shoots (1.4–1.8-fold), and Mg increased in both roots (1.4–1.7-fold) and shoots (1.2–1.7-fold). ANE A further enhanced nutrient content, following the order 1/10 MS + ANE A > 1/10 MS Control > 0 MS + ANE A > 0 MS Control. Foliar application was generally more effective, especially for K (roots +55–74%; shoots +24%, p ≤ 0.001) compared with root application (roots +37–40%; shoots +16%, p ≤ 0.005). Unlike barley, Ca showed only small changes in wheat, with a slight but significant shoot increase after foliar application (+8%, p ≤ 0.05). Both application modes enhanced P and Mg, but significant effects were more consistent for foliar treatment (roots: P + 12%, p ≤ 0.05; Mg +25%, p ≤ 0.001; shoots: P + 8%, Mg +9%, p ≤ 0.05–0.01). Root application produced smaller gains, significant only for P in roots (+25%, p ≤ 0.05) and Mg in shoots (+10%, p ≤ 0.05).

In summary, barley was more responsive to K and Ca enrichment, while wheat was more responsive to K, Ca, P, and Mg uptake under reduced nutrient regime. Foliar ANE A application consistently promoted stronger root K uptake in both crops along with higher Ca and P in root in barley. Foliar ANE A boosted P and Mg in both tissues in wheat. However, root ANE A application showed more nutrient-specific and tissue-dependent effects.

### 3.6 Effect of nutrient conditions and ANE A on micronutrient content of barley and wheat

Micronutrient content was generally higher in 1/10 MS than 0 MS conditions. In barley seedlings, 1/10 MS increased Mn and Mo in both tissues, Cu and Zn in shoots, and Fe in roots (p ≤ 0.001; **Supplementary Tables 7**, **S8**). Foliar application of ANE A further stimulated root B (+15%, p ≤ 0.001), shoot Fe (+26%, p ≤ 0.001), and Zn in both tissues (+11–14%, p ≤ 0.01), with significant nutrient regime × biostimulant interactions for roots with Mn, Cu, Fe, Mo and shoots for B, Cu, Mo. Under 0 MS, foliar treatment produced the strongest effects, increasing root B (+43%), Mn (+17%), Cu (+43%), Fe (+66%), and Mo (+1.1 ng) as well as shoot Cu (+17%) and Mo (+71%) (all p ≤ 0.001). In 1/10 MS, the foliar effect was more limited, enhancing only root Mn (+16%, p ≤ 0.01) and Mo (+37%, p ≤ 0.001) and shoot Mo (+21%, p ≤ 0.001), while slightly decreasing shoot Cu (−9%, p ≤ 0.001) (**Supplementary Table 7**). ANE A root application produced smaller responses, increasing shoot Mn (+7%, p ≤ 0.05), root Cu (+18%, p ≤ 0.05), and root Mo (+13%, p ≤ 0.05). Zn shifts depended on nutrient regime: in 0 MS, root Zn rose (+3%) while shoot Zn declined (−5%); the opposite occurred in 1/10 MS (root −12%, shoot +6%) (all p ≤ 0.05) (**Supplementary Table 8**).

Wheat seedlings also accumulated micronutrients under 1/10 MS, particularly Mn, Fe, Mo, and Zn in both tissues, and Cu and B in shoots (p ≤ 0.05; **Supplementary Tables 9**, **S10**). Foliar ANE A application significantly increased all micronutrients tested except shoot Mn. Consistent responses across nutrient regimes included higher root Cu (+28%), Zn (+19%), shoot Zn (+16%), and shoot Fe (+17%), alongside reduced root Fe (−11%) (all p ≤ 0.01–0.001). Wheat was more responsive under 1/10 MS, with marked increases in root B (+11%), Mn (+36%), shoot Cu (+31%), and root and shoot Mo (+36%) (all p ≤ 0.001). By contrast, in 0 MS, foliar application reduced B (root −50%, shoot −17%) and shoot Cu (−3%), while enhancing root Mn (+19%) and shoot Mo (+66%) (all p ≤ 0.001) (**Supplementary Table 9**). ANE A root application had a weaker overall effect but was more pronounced under 1/10 MS, increasing root Mn (+31%), Cu (+38%), shoot Fe (+3.8-fold), and root/shoot Mo (+27% and +70%, all p ≤ 0.001). In 0 MS, it led to smaller increases in root Mn (+4%), Cu (+22%), shoot Fe (+16%), and root Mo (+44%) but reduced shoot Mo (−77%) (all p ≤ 0.001) (**Supplementary Table 10**).

Overall, ANE A modulated micronutrient dynamics in a crop- and nutrient-regime–dependent manner. In barley, foliar application was more effective under nutrient deprivation (0 MS), particularly for Cu, Fe, and Mo, while in wheat, stronger responses were observed under reduced nutrient supply (1/10 MS), especially for Mn, Mo, and Cu. Root application produced more moderate but consistent improvements in both crops, with greater relative effects under 1/10 MS.

### 3.7 Effect of ANE A on NUE markers of barley and wheat

Significant nutrient regime × biostimulant interactions were observed in several nutrient content determinations in barley and wheat. Experiments conducted in 0 MS medium demonstrated that ANE A promotes nutrient relocation from the seed to the seedling, as no external nutrients were available in the growth medium. These findings indicated that comparison of NUE markers between control and ANE A treated plants grown under reduced nutrient supply (1/10 MS) had to be performed considering the effect of the background without the addition of any fertiliser (0 MS).

**Figure 3:**
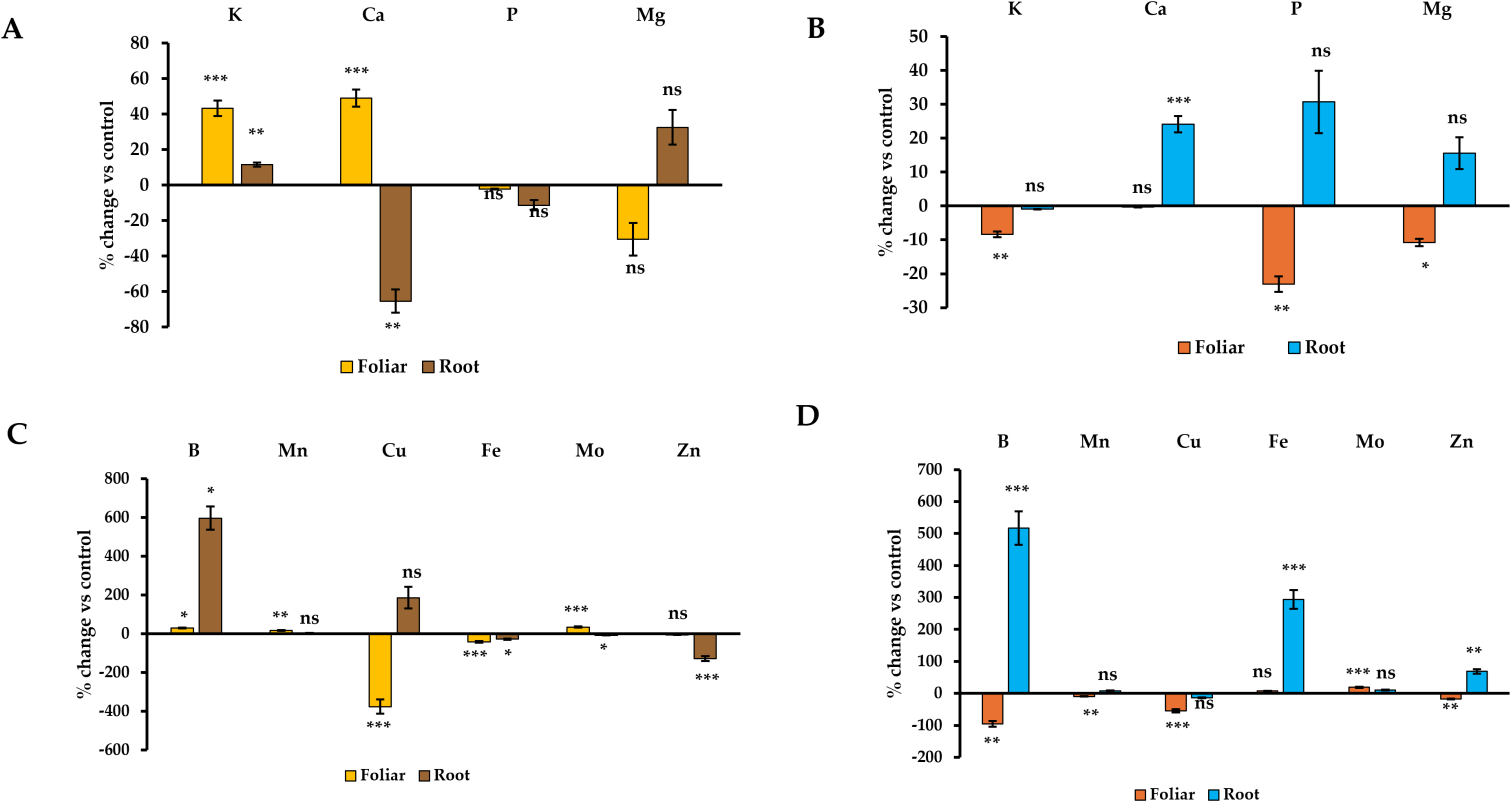
Effect of ANE A treatments on RE of Barley root and shoot tissues. Nutrients change in root **(A, C)** and shoot tissues **(B, D)**. The figure legends refer to method of ANE A application. All data are expressed as percentage change with respect to the control per root/shoot sample in 12-day old plant seedlings. ns, *, **, and *** means non-significant or significant at *p* ≤ 0.05, *p* ≤ 0.01, and *p* ≤ 0.001, respectively. Different letters indicate statistical differences with *p* ≤ 0.05 based on t-test between control and ANE A ANE treatment for each tissue and application mode. Number of biological replicates (n ≥ 3).

The RE calculation measures how much of the applied nutrient was taken up by the plant tissue, with a positive value meaning that the crop has absorbed part of the nutrient and a negative value suggesting that the crop took up less nutrient compared to the respective 0 MS medium sample. As observed in **Figure 3**, foliar application of ANE A in barley increased root RE but decreased the respective shoot value for K (+43% and -8%, p ≤ 0.01), B (+28% and -95%, p ≤ 0.05), and Mn (+14% and -9%, p ≤ 0.01). However, RE increased for Mo in both tissues (+33% and +19%, p ≤ 0.001) and Ca in root tissue (+49%, p ≤ 0.001). In contrast, this uptake efficiency marker decreased significantly in shoot tissue for P, Mg and Zn (-23%, -11% and -17%, p ≤ 0.05) and, especially for Cu in root and shoot tissues (-3.8-fold and -54%, p ≤ 0.001). This negative result was concordant with the stronger response of the biostimulant under 0 MS medium (**Supplementary Table 8**). ANE A root applied decreased RE in root but increased in shoot for Ca (-65% and +24%, p ≤ 0.01), Fe (-29% and +2.9-fold, p ≤ 0.05) and Zn (-1.3-fold and +69%, p ≤ 0.01). A remarkably higher RE was also observed in both root and shoot for B (+5.9-fold and +5.2-fold, p ≤ 0.05), a moderate increase for root K (+11%, p ≤ 0.01) and a slight decrease for root Mo (-8%, p ≤ 0.05). Overall, the mode of application of ANE A seems to differently modulate nutrient uptake efficiency in barley seedlings, with foliar and root application being more effective in root RE and shoot RE values, respectively (**Figure 3**).

**Figure 4:**
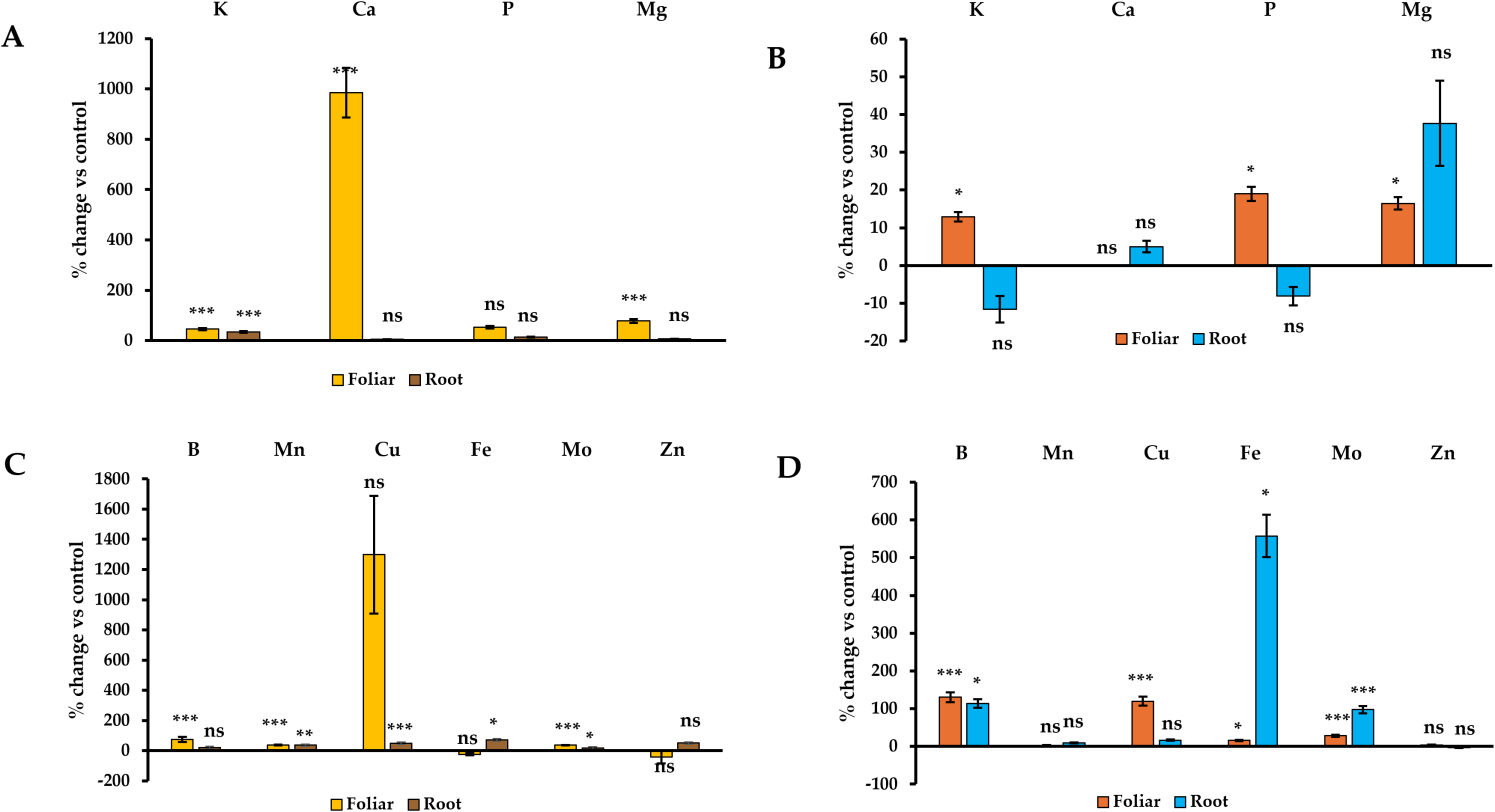
Effect of ANE A treatment on RE of wheat root and shoot tissues. Nutrients change in root **(A, C)** and shoot tissues **(B, D)**. The figure legends refer to method of ANE A application. All data are expressed as percentage change with respect to the control per root/shoot sample in 12-day old plant seedlings. ns, *, **, and *** means non-significant or significant at *p* ≤ 0.05, *p* ≤ 0.01, and *p* ≤ 0.001, respectively. Different letters indicate statistical differences with *p* ≤ 0.05 based on t-test between control and ANE A. ANE treatment for each tissue and application mode. Number of biological replicates (n ≥ 3).

Foliar application of ANE A in wheat seedlings had a significant effect in improving RE in root and shoot of several nutrients such as K (+46% and +13%, p ≤ 0.05), Mg (+78% and +16%, p ≤ 0.05), B (+118% and +130%, p ≤ 0.001) and Mo (+36% and +28%, p ≤ 0.001). The biostimulant also increased RE from Ca and Mn in root tissue (9.9-fold, +38%, p ≤ 0.001) and P, Cu and Fe in shoot tissue (+18%, +119% and +16%, respectively, p ≤ 0.05). ANE A root application had a positive overall effect in certain nutrients, improving RE in root and shoot of Fe (+71% and +5.5-fold, p ≤ 0.05) and Mo (+19% and +97%, p ≤ 0.05). A significant enhancement of root uptake efficiency of K (+34%, p ≤ 0.001), Mn (+36%, p ≤ 0.01) and Cu (+50%, p ≤ 0.001), along with a remarkably improvement in RE for B in shoot (+114%, p ≤ 0.05) was also observed. These results indicate that ANE A can enhance nutrient uptake more effectively in wheat than in barley, particularly when applied via foliar application (**Figure 4**).

AE determined the improvement in tissue biomass resulting from the respective nutrient input. Barley seedlings treated with ANE A by foliar spray had significantly higher AE for all nutrients in the roots (+41%, p ≤ 0.01), but significantly lower AE in the shoots (-13%, p ≤ 0.05). When the biostimulant was root applied, a larger increase was observed in AE for all nutrients in barley roots by +89% (p ≤ 0.05), while a small non-statistically significant decrease was observed in the shoot values (-3.7%, p = 0.814).

AE analysis for wheat seedlings showed that foliar application of ANE A stimulated a significant increase of +39% (p ≤ 0.05) in AE values of all nutrients analysed, with no numerical difference detected in the shoot values. Root application of ANE A did show a similar pattern as the observed in barley, with higher AE values for root tissue +45% (p ≤ 0.05), with a small decrease in shoot (-3.4%, p = 0.759).

Taken together, NUE marker assessment demonstrates that ANE A can enhance both nutrient uptake and agronomic efficiency in both barley and wheat for key nutrients, with a stronger positive effect in wheat. Likewise tissue-specific and application-method-dependent responses must be considered when integrating ANE A into crop nutrient management strategies.

## 4 Discussion

In an era when plant productivity is increasingly threatened by abiotic stresses, nutrient deficiencies, soil erosion and pollution, there is a growing need to evaluate the actual effects of plant biostimulants, as well as the corroboration of their claimed benefits, and the identification of reliable metrics to assess their impact (Du Jardin et al., 2025).

Early seedling development is the stage at which crops are the least resilient and most susceptible to losses caused by environmental pressure, both in the presence and absence of nutrients. The first objective of this study was to assess the effect of three different ANE biostimulants on wheat whole seedlings biomass and nutrient contents. The three ANE products were standardized based on total solids content (5 mg per treatment) to ensure that comparisons reflected intrinsic biological activity rather than differences in formulation concentration. Due to variation in manufacturing processes, this resulted in different field-equivalent application rates, and this approach was intentionally adopted to enable a direct comparison of extract potency on a per-unit-active basis.

The second objective of this study was to evaluate the impact of the most responsive ANE biostimulant (ANE A) applied either as foliar spray or via direct root application, on NUE in two important cereal crops (barley and wheat) grown *in vitro*. Nutrient uptake was assessed in both root and shoot tissues under nutrient deprivation (0 MS) and reduced nutrient supply (1/10 MS). Unlike most previous studies, which have focused on ANE effects primarily on stress tolerance, nitrogen uptake, or yield traits at later developmental stages, the present work delivers the first comprehensive evaluation of multiple essential nutrients (macro-, secondary, and micronutrients) within a single, coherent experimental framework. The use of a strictly controlled *in vitro* agar-based system enabled the isolation of direct biostimulant effects on plant nutrient dynamics by minimizing confounding influences such as soil heterogeneity, microbial interactions, and variable nutrient availability. By examining nutrient uptake and partitioning dynamics in barley and wheat seedlings under strictly controlled *in-vitro* conditions, independent of soil or microbial interactions, this study provides a novel mechanistic perspective on the role of biostimulants during early crop establishment. While such a reductionist system does not fully replicate soil-based agricultural environments, it provides a robust framework for mechanistic interpretation, which is often difficult to achieve under field conditions. The integration of tissue-specific nutrient profiling with nutrient use efficiency (NUE) indices enables direct linkage between nutrient acquisition and biomass production, offering a more functional interpretation of biostimulant action. However, the extrapolation of these findings to field conditions should be made with caution, as soil systems introduce additional complexity including plant–microbe interactions, nutrient sorption dynamics, and physical constraints on root growth.

### 4.1 Comparative assessment of different ANEs on phenotypic and nutritional contents in wheat seedlings

During the first screening of the three ANEs, ANE A and C were able to improve the total biomass of the wheat seedlings. While both ANE A and ANE C significantly increased total biomass relative to the control (+22.7% and +21.0%, respectively), ANE A induced a significantly greater increase in root biomass (+41.1%), which was statistically higher than both ANE B and ANE C. The effect of ANE A was mostly on the root biomass while ANE C was more effective on the shoot biomass. All three ANEs were able to improve the content of K, Ca, P, Mg and Mn in wheat seedlings. Additionally, ANE A and ANE C increased the Mo content. However, only ANE A was able to increase B and Zn contents. Nutrient profiling further demonstrated that ANE A produced the most comprehensive enhancement, significantly increasing eight essential nutrients (K, Ca, P, Mg, B, Mn, Mo, and Zn), compared to five for ANE B and six for ANE C, confirming its broader impact on plant nutritional status. Previously, it has been shown that the application of seaweed extracts can improve these nutrients in different crops (Abou El-Yazied et al., 2012; Billard et al., 2014; Carrasco-Gil et al., 2018; Ertani et al., 2018; Elsharkawy et al., 2021).

Although the compositional profile of ANE A overlapped with ANE B in four compositional parameters (ash, laminarin, free mannitol and polyphenols), and none with ANE C (**Table 1**), A and C exhibited the same effectiveness in biomass production when applied at the same dose. Indicating that different ANEs act differently (Goñi et al., 2016), and the efficiency should be attributed to concentration and the size of the active molecules rather than total content of given fraction. Overall, ANE A showed the most robust performance in terms of biomass production and NUE, thus was selected for further studies. These results highlight that extract composition alone does not fully predict biological efficacy, reinforcing the importance of functional screening in biostimulant evaluation.

### 4.2 Impact of ANE A on phenotypic markers of barley and wheat seedlings

The root is one vital component that is crucial for plant growth and development, providing plants with water, nutrients and mechanical support, which are linked to the development of higher crop yield (Fageria and Moreira, 2011; Sathiyavani et al., 2017). The application of ANE A to barley and wheat seedlings, either via foliar spray or direct root application, was able to increase root biomass significantly, irrespective of the nutrient regime (absent or reduced supply). These findings are in line with Goñi et al., 2021, who found that the application of another alkaline ANE (PSI-362) increased barley seedling biomass when it was applied by foliar spray or coated with fertiliser under reduced N fertiliser rate. A clear difference in biomass growth was also observed in wheat seedlings treated with the same ANE biostimulant PSI-362 when it was grown under nutrient starvation conditions (0 MS) and reduced nutrient supply (1/10 MS) (Łangowski et al., 2022). Our results are also supported by Shukla and Prithiviraj (2021) where they found that ANE improved fresh and dry weight of roots and shoots of maize under phosphorus limiting conditions. Additionally, Mutlu‐Durak et al., 2024 found that brown seaweed extracts (SWEs) were able to enhance the root biomass of wheat seedling. Strong root growth in the early stages is paramount for efficient nutrient and water uptake throughout the plant’s life cycle (Wang et al., 2016). Although a slight increase was observed in the shoot biomass of barley and wheat when ANE A was applied via foliar spray, a significant increase was seen in the shoot of wheat seedling treated with ANE A via root application. Indicating further the growth-promoting abilities of ANE A, however the application manner may have different outcomes in different crops most likely due to different abilities to induce plant signalling after molecule perception by the plant.

### 4.3 Impact of ANE A on macro, secondary and micronutrients

Testing ANE A under nutrient starvation (0 MS) and reduced nutrient supply (1/10 MS) demonstrated that in both wheat and barley the product not only enhanced nutrient uptake from the medium but also facilitated nutrient remobilization from the seed into seedling tissues. This interpretation is supported by the observed increases in total nutrient content at the whole-plant level (combined root and shoot) under 0 MS conditions, where no external nutrient supply was available, indicating that these increases most likely originate from enhanced mobilization and redistribution of internal seed reserves. Seedlings germinated in nutrient free medium (0 MS) relied solely on seed reserves (Welch, 2024), whereas seedlings grown in 1/10 MS used both endogenous and external nutrients. Under these conditions, a plausible explanation for increased tissue nutrient content is enhanced mobilization and translocation of nutrients from internal seed reserves. This external nutrient supply strongly shapes the mineral profile of barley and wheat seedlings, reinforcing the direct relationship between nutrient availability and internal concentrations (Marschner, 2012). Against this background, ANE A treatment produced significant, crop-specific changes in both nutrient profiles, supporting its role as a modulator of nutrient uptake and remobilization. Nutrient uptake is tightly regulated and influenced by both internal status and environmental availability. Given ANE A’s complex composition and the diversity of nutrients assessed, consistent patterns across all elements were not expected. Nevertheless, meaningful insights can be drawn for individual nutrients.

The most consistent effects were observed for potassium (K) and phosphorus (P). K, essential for osmotic balance and root development (Wang et al., 2013; Sustr et al., 2019), was elevated in both roots and shoots under both 0 MS and 1/10 MS, with wheat showing strong increases even under nutrient deprivation—suggesting mobilization from seed reserves. Similar stimulation of K uptake has been reported for seaweed extracts (Billard et al., 2014; Elsharkawy et al., 2021). P, central to early root vigour (Zhang et al., 2016), also increased across most tissues and conditions.

Calcium (Ca) and magnesium (Mg) responses were more nuanced. Ca, important for membrane stability and stress signalling (Parvin et al., 2019), increased primarily in shoots despite occasional declines in roots, indicating enhanced translocation. Mg, vital for photosynthesis and ROS control (Mengutay et al., 2013), increased consistently, with foliar applications favouring root accumulation and root applications enhancing shoot levels. These results highlight how ANE A application mode influences nutrient partitioning.

Micronutrient responses to ANE A were strongly species and tissue dependent. In barley, foliar application enhanced root B, shoot Fe, and root/shoot Zn, while root application stimulated Mn and Mo. In wheat, root Cu and shoot Fe and Zn were consistently elevated. Increases in Fe and Zn, both key to antioxidant defence and energy metabolism (Bradáčová et al., 2016; Al-Amri et al., 2020), are especially relevant for seedling vigour. Mn responses varied: foliar ANE A increased barley shoot Mn, while wheat roots responded most under 1/10 MS. Mo increases under 1/10 MS reflect its role in enzymatic activity (Kaiser et al., 2005), while B responses suggest altered partitioning consistent with a previous brown SWEs study (Ertani et al., 2018).

Clear contrasts emerged between the two cereals. In barley, responses were largely root-focused: foliar application enhanced K, Ca, B, Mn, and Mo in roots, while root application stimulated Ca, B, Fe, Zn, and Mo in shoots, suggesting some xylem-driven transport but limited systemic redistribution. This root-dominant strategy aligns with barley’s adaptation to stress-prone environments (Abbas et al., 2011). In wheat, both roots and shoots benefited more broadly. ANE A foliar treatments increased K, Mn, Cu, Fe, Mo, and P, while root application stimulated multiple macro- and micronutrients. Wheat’s stronger shoot responses indicate efficient systemic redistribution, integrating uptake across tissues (Anbessa and Juskiw, 2012; Huang et al., 2023). Simultaneous increases in mobile (K, P, Mg) and immobile (B, Fe, Cu, Mo) nutrients highlight ANE A’s potential to promote synergistic nutrient interactions in wheat seedlings (Roy et al., 2022; Ganie et al., 2013).

Overall, ANE A had the strongest and most consistent effects on mobile nutrients (K, P, Mg, Mo), while effects on less mobile elements (Ca, Fe, Zn, Cu, Mn, B) were more variable and tissue specific. This reflects inherent differences in nutrient mobility and species-specific transport constraints. Barley relied more on root-driven acquisition, whereas wheat exhibited balanced systemic enhancement. The simultaneous increases in mobile and immobile nutrients, particularly in wheat, point to synergistic nutrient interactions (Roy et al., 2022; Ganie et al., 2013). Taken together, these results suggest that ANE A is a powerful stimulator of macro, secondary and micronutrient use efficiency, with outcomes shaped by crop physiology, nutrient mobility, and the route of application.

### 4.4 Impact of ANE A on nutrient recovery and agronomic efficiency

Nutrient use efficiency (NUE) is a critical determinant of agricultural productivity, reflecting how effectively plants acquire, translocate, and utilize available nutrients (Dobermann, 2007; Fixen et al., 2015). Although NUE indices such as Recovery Efficiency (RE) and Agronomic Efficiency (AE) are traditionally applied in field-based fertilizer studies, their underlying mathematical framework is general and can be adapted to controlled experimental systems. In this study, NUE was evaluated in terms of recovery efficiency (RE) and agronomic efficiency (AE), providing complementary insights into nutrient uptake dynamics and biomass productivity in response to ANE A application. To enable this approach, a defined nutrient gradient was established using 0 MS (nutrient-free) and 1/10 MS (reduced-nutrient) media, allowing RE (U – U_0_) and AE (Y – Y_0_) to be calculated relative to a controlled baseline. In this context, these indices are used as comparative analytical tools to quantify treatment effects on nutrient acquisition and biomass production, rather than as direct analogues to field-derived NUE values.

The effects of ANE A were strongly influenced by both plant species and application method, with wheat showing a broader and more consistent improvement in nutrient uptake efficiency across both root and shoot tissues than barley. In barley, foliar ANE A application significantly increased RE in roots for K, Ca, B, Mn, and Mo, but untreated controls often exhibited higher RE for several nutrients in shoots, including K, P, Mg, B, Mn, Cu, and Zn. This suggests that while ANE A enhances nutrient acquisition at the root level, it may also alter translocation patterns, potentially favouring nutrient retention in roots over transfer to shoots. The pronounced increases in Ca, B, Fe, and Zn RE in the shoots of root-applied ANE A barley further support the idea of application-method-dependent shifts in nutrient partitioning (Marschner, 2012) driven by tissue specific biostimulant molecule perception and signalling.

In wheat, ANE A enhanced RE as foliar or root applied for a wider range of nutrients in both tissues, including substantial increases for K, Ca, Mg, B, Mn, and Mo in roots, and K, P, Mg, B, Cu, Fe, and Mo in shoots. The exceptionally high increases in Ca RE in roots (+9.9-fold) and Fe RE in shoots (+5.5-fold) highlight a potentially strong influence of ANE A on nutrient-specific transport pathways. This greater responsiveness in wheat may be due to species-specific root architecture, nutrient demand, or genetic makeup determining receptor and physiological sensitivity to the bioactive compounds present in ANE A.

AE patterns further underline these species differences. In both crops, ANE A improved this NUE marker in roots (+41% in barley, +39% in wheat) but had limited or reduced accumulation in shoots. This tissue-specific divergence suggests that enhanced nutrient recovery in roots does not always translate into proportional gains in shoot biomass, potentially due to altered allocation strategies or delayed nutrient remobilisation to aboveground tissues during early seedling growth.

The molecular mechanisms underlying these effects likely involve changes in nutrient uptake and transport systems. However, it should be noted that no direct molecular analyses (e.g., gene expression or protein activity measurements) were performed in this study. Previous studies have shown that ANEs can upregulate genes encoding key nutrient transporters, thereby improving acquisition of both macro and micronutrients (Goñi et al., 2016; Shukla et al., 2019; El Boukhari et al., 2020; Goñi et al., 2021; Shukla and Prithiviraj, 2021; Łangowski et al., 2022). For example, genes such as *HAK5* and *AKT1* (K uptake), *PHT1;1* and *PHO1* (P uptake), CAX1 and CDPKs (Ca), IRT1 and FRO2 (Fe), MOT1 (Mo), ZIP1 and HMA4 (Zn), BOR1 and NIP5;1 (B), COPT1/2 (Cu), and NRAMP1 (Mn) may be involved in the observed nutrient-specific responses (Reid and Hayes, 2003; Zelazny and Vert, 2014). While such regulatory effects were not directly evaluated here, the observed increases in nutrient accumulation and RE are therefore interpreted at a physiological level and are consistent with, but do not confirm, modulation of nutrient transporter activity, and should therefore be interpreted as a plausible, literature-supported hypothesis rather than a demonstrated mechanism. ANEs are complex mixtures of bioactive molecules including well-known carbohydrates such as alginate, fucoidan and laminarin that can exert multifaceted effects on plant metabolism (Goñi et al., 2016, 2020; El Boukhari et al., 2020). The differential species responses observed here suggest that ANE A’s bioactivity interacts with inherent physiological traits to determine nutrient acquisition and allocation outcomes. This points to the potential for crop-specific optimisation of ANE A application strategies (e.g., foliar vs root, timing, dose) to maximise NUE benefits.

Overall, these findings demonstrate that ANE A has significant potential to enhance uptake and translocation of key macro, secondary and micronutrients in cereal seedlings, with wheat appearing more responsive than barley in both root and shoot tissues. While these results provide a strong mechanistic proof-of-concept under controlled conditions, further validation under soil and field environments is required to confirm their agronomic relevance, particularly under standardized field application rates. Future work should combine physiological measurements with transcriptomic and transporter activity assays to clarify the molecular basis of these responses, assess their persistence under field conditions, and evaluate whether similar improvements in NUE can be achieved across diverse genotypes and soil nutrient regimes.

## 5 Conclusions

In conclusion, growth and nutrient status of both barley and wheat seedlings were greatly enhanced by the application of nutrients (1/10 MS) confirming the fundamental requirement for adequate nutrition. ANE biostimulants influence nutrient uptake but the profile of nutrient changes and impact on seedling biomass growth is different between different ANE biostimulant products. Importantly, the biostimulant ANE A consistently and significantly increased root biomass in both crops, which is essential for early establishment and nutrient uptake. Furthermore, ANE A increased nutrient acquisition and recovery efficiency, especially in wheat, by modulating the absorption and distribution of a broad range of macro, secondary and micronutrients, frequently exhibiting synergistic effects with nutrient treatment. These effects are interpreted as physiological responses observed under controlled conditions and provide a mechanistic basis for improved nutrient use efficiency, rather than direct evidence of specific molecular pathways. The complex and dynamic mechanisms through which biostimulants function are highlighted by the observed interactions between ANE A and nutrient levels, which may play a part in optimising plant physiological processes for improved NUE. Future research could focus on elucidating the precise molecular and physiological mechanisms underlying the effects of ANE A, especially its role in root development and differential nutrient partitioning in cereal seedlings. Future research should include validation under soil and field conditions, as well as integration with molecular and physiological approaches, to confirm the mechanisms and agronomic relevance of these responses.

## Data Availability

The original contributions presented in the study are included in the article/**Supplementary Material**. Further inquiries can be directed to the corresponding author.

## Supplementary material

Supplementary Figure 1 Effect of nutrient conditions and ANE A application on barley and wheat seedlings. A red dashed line has been drawn to separate visually the root and aboveground tissues. Scale of background square equals 2 cm H X by 2 cm W.
