# Supplementary material for: Impact of Ascophyllum nodosum extract biostimulants on nutrient use efficiency and seedling establishment in wheat and barley
Source: Front Plant Sci. 2026 May 12;17:1813433. doi: 10.3389/fpls.2026.1813433 (PMC13201462; doi:10.3389/fpls.2026.1813433)
Supplement: Supplementary Figure 1 — Effect of nutrient conditions and ANE A application on barley and wheat seedlings. A red dashed line has been drawn to separate visually the root and aboveground tissues. Scale of background square equals 2 cm H X by 2 cm W. [file DataSheet1.docx]

Supplementary Material

**
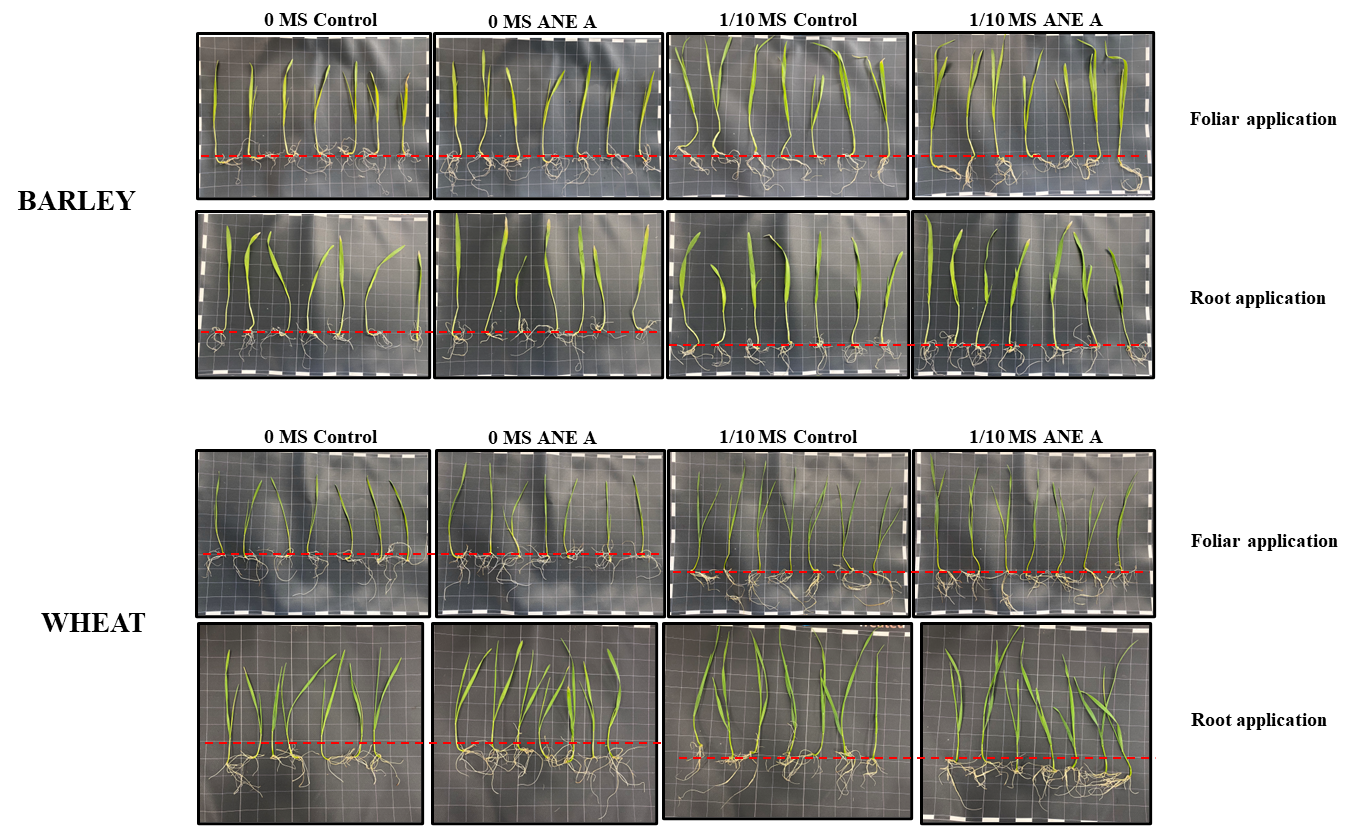
**

Figure S1. Effect of nutrient conditions and ANE A application on barley and wheat seedlings. A red dashed line has been drawn to separate visually the root and aboveground tissues. Scale of background square equals 2 cm H X by 2 cm W.

**Table S1.** Effect of nutrient conditions and foliar application of ANE A on moisture content of barley and wheat

| **Source of variance** | **Barley** | | **Wheat** | | |
| --- | --- | --- | --- | --- | --- |
|  | **Root**  **% Moisture** | **Shoot**  **% Moisture** | **Root**  **% Moisture** | **Shoot**  **% Moisture** | |
| **Nutrient (N)** |  |  |  |  | |
| **0 MS** | 87.47 | 87.76 a | 83.56 | 84.97 a | |
| **1/10 MS** | 90.45 | 90.75 b | 87.78 | 87.41 b | |
| **Biostimulant (B)** |  |  |  |  | |
| **Control** | 88.38 | 89.32 | 85.30 | 85.97 | |
| **ANE A** | 89.55 | 89.19 | 86.05 | 86.42 | |
| **N X B** |  |  |  |  | |
| **0 MS x Control** | 87.12 | 87.83 | 82.76 | 84.67 | |
| **0 MS x ANE A** | 87.83 | 87.68 | 84.35 | 85.27 | |
| **1/10 MS x Control** | 89.64 | 90.80 | 87.83 | 87.26 | |
| **1/10 MS x ANE A** | 91.27 | 90.69 | 87.74 | 87.56 | |
| **Statistical significance** | | | | |  |
| **Nutrient (N)** | **ns** | ****** | **ns** | ***** | |
| **Biostimulant (B)** | **ns** | **ns** | **ns** | **ns** | |
| **N × B** | **ns** | **ns** | **ns** | **ns** | |

All data are expressed as average per sample in 12-day old plant seedlings. ns, *, **, and *** means non-significant or significant at *p* ≤ 0.05, *p* ≤ 0.01, and *p* ≤ 0.001, respectively. Different letters indicate statistical differences with *p* ≤ 0.05 based based on t-test for N factor. Number of biological replicates (n ≥ 15).

**Table S2.** Effect of nutrient conditions and root application of ANE A on moisture content of barley and wheat

| **Source of variance** | **Barley** | | **Wheat** | | |
| --- | --- | --- | --- | --- | --- |
|  | **Root**  **% Moisture** | **Shoot**  **% Moisture** | **Root**  **% Moisture** | **Shoot**  **% Moisture** | |
| **Nutrient (N)** |  |  |  |  | |
| **0 MS** | 85.73 a | 87.51 a | 87.58 a | 84.42 a | |
| **1/10 MS** | 88.10 b | 89.55 b | 89.93 b | 86.82 b | |
| **Biostimulant (B)** |  |  |  |  | |
| **Control** | 86.38 | 88.37 | 88.51 | 85.46 a | |
| **ANE A** | 87.46 | 88.70 | 89.01 | 85.79 b | |
| **N X B** |  |  |  |  | |
| **0 MS x Control** | 85.21 | 87.27 | 87.23 | 84.20 | |
| **0 MS x ANE A** | 86.26 | 87.85 | 87.94 | 84.64 | |
| **1/10 MS x Control** | 87.55 | 89.56 | 89.78 | 86.71 | |
| **1/10 MS x ANE A** | 88.66 | 89.55 | 90.09 | 86.94 | |
| **Statistical significance** | | | | |  |
| **Nutrient (N)** | ***** | ***** | ***** | ******* | |
| **Biostimulant (B)** | **ns** | **ns** | **ns** | ***** | |
| **N × B** | **ns** | **ns** | **ns** | **ns** | |

All data are expressed as average per sample in 12-day old plant seedlings. ns, *, **, and *** means non-significant or significant at *p* ≤ 0.05, *p* ≤ 0.01, and *p* ≤ 0.001, respectively. Different letters indicate statistical differences with *p* ≤ 0.05 based based on t-test for N and B factors. Number of biological replicates (n ≥ 15).

Table S3. Effect of nutrient conditions and foliar application of ANE A on macro- and secondary nutrient content in barley tissues

| **Source of variance** | **Content K (µg)** | | **Content Ca (µg)** | | **Content P (µg)** | | | **Content Mg (µg)** | |
| --- | --- | --- | --- | --- | --- | --- | --- | --- | --- |
|  | **Root** | **Shoot** | **Root** | **Shoot** | **Root** | | **Shoot** | **Root** | **Shoot** |
| **Nutrient (N)** |  |  |  |  |  | |  |  |  |
| **0 MS** | 100 a | 910 a | 8.8 a | 24.1 a | 57.9 a | | 169.0 a | 14 a | 56 a |
| **1/10 MS** | 490 b | 2790 b | 24.7 b | 75.0 b | 97.5 b | | 239.0 b | 16 b | 87 b |
| **Biostimulant (B)** |  |  |  |  |  | |  |  |  |
| **Control** | 240 a | 1760 a | 14.9 a | 50.1 | 73.9 a | | 207.0 | 14 a | 71 |
| **ANE A** | 350 b | 1930 b | 18.6 b | 49.1 | 79.6 b | | 201.0 | 16 b | 72 |
| **N X B** |  |  |  |  |  | |  |  |  |
| **0 MS x Control** | 81 a | 780 | 8.5 a | 24.5 | 54.7 | | 168.0 | 13 | 54 |
| **0 MS x ANE A** | 120 a | 1030 | 9.0 a | 23.6 | 61.0 | | 170.0 | 15 | 58 |
| **1/10 MS x Control** | 400 b | 2750 | 21.3 b | 75.6 | 93.1 | | 247.0 | 15 | 87 |
| **1/10 MS x ANE A** | 580 c | 2830 | 28.2 c | 74.5 | 98.3 | | 231.0 | 16 | 87 |
| **Statistical significance** | | | | | | | | | |
| **Nutrient (N)** | ******* | ******* | ******* | ******* | ******* | | ******* | ****** | ******* |
| **Biostimulant (B)** | ******* | ****** | ******* | **ns** | ***** | | **ns** | ***** | **ns** |
| **N × B** | ******* | **ns** | ******* | **ns** | **ns** | **ns** | | **ns** | **ns** |

All data are expressed as average per sample in 12-day old plant seedlings. ns, *, **, and *** means non-significant or significant at *p* ≤ 0.05, *p* ≤ 0.01, and *p* ≤ 0.001, respectively. Different letters indicate statistical differences with *p* ≤ 0.05 based on t-test for N and B factors and one-way ANOVA for N x B factor. Number of biological replicates (n ≥ 4).

Table S4. Effect of nutrient conditions and root application of ANE A on macro- and secondary nutrient content in barley tissues

| **Source of variance** | **Content K (µg)** | | **Content Ca (µg)** | | **Content P (µg)** | | | **Content Mg (µg)** | |
| --- | --- | --- | --- | --- | --- | --- | --- | --- | --- |
|  | **Root** | **Shoot** | **Root** | **Shoot** | **Root** | | **Shoot** | **Root** | **Shoot** |
| **Nutrient (N)** |  |  |  |  |  | |  |  |  |
| **0 MS** | 75 a | 330 a | 8.1 a | 8.9 a | 67 a | | 141 a | 14 | 42 a |
| **1/10 MS** | 220 b | 820 b | 15.7 b | 20.7 b | 85 b | | 268 b | 13 | 54 b |
| **Biostimulant (B)** |  |  |  |  |  | |  |  |  |
| **Control** | 130 a | 510 a | 13.6 b | 13.7 a | 73 a | | 150 a | 13 | 46 a |
| **ANE A** | 160 b | 640 b | 10.1 a | 15.8 b | 79 b | | 159 b | 14 | 50 b |
| **N X B** |  |  |  |  |  | |  |  |  |
| **0 MS x Control** | 62 | 260 | 8.0 | 8.4 a | 63 | | 138 | 14 | 40 |
| **0 MS x ANE A** | 87 | 400 | 8.1 | 9.3 a | 71 | | 144 | 14 | 44 |
| **1/10 MS x Control** | 200 | 750 | 19.3 | 19.0 b | 83 | | 162 | 13 | 51 |
| **1/10 MS x ANE A** | 240 | 880 | 12.1 | 22.3 c | 88 | | 175 | 14 | 56 |
| **Statistical significance** | | | | | | | | | |
| **Nutrient (N)** | ******* | ******* | ******* | ******* | ******* | | ******* | **ns** | ******* |
| **Biostimulant (B)** | ******* | ******* | ***** | ******* | ***** | | ***** | **ns** | ****** |
| **N × B** | **ns** | **ns** | **ns** | ******* | **ns** | **ns** | | **ns** | **ns** |

All data are expressed as average per sample in 12-day old plant seedlings. ns, *, **, and *** means non-significant or significant at *p* ≤ 0.05, *p* ≤ 0.01, and *p* ≤ 0.001, respectively. Different letters indicate statistical differences with *p* ≤ 0.05 based based on t-test for N and B factors and one-way ANOVA for N x B factor. Number of biological replicates (n ≥ 4).

Table S5. Effect of nutrient conditions and foliar application of ANE A on macro- and secondary nutrient content in wheat tissues

| **Source of variance** | **Content K (µg)** | | **Content Ca (µg)** | | **Content P (µg)** | | **Content Mg (µg)** | | |
| --- | --- | --- | --- | --- | --- | --- | --- | --- | --- |
|  | **Root** | **Shoot** | **Root** | **Shoot** | **Root** | **Shoot** | **Root** | **Shoot** | |
| **Nutrient (N)** |  |  |  |  |  |  |  |  | |
| **0 MS** | 140 a | 380 a | 9.2 a | 9.99 a | 104.0 b | 84.7 a | 8 a | 16 a | |
| **1/10 MS** | 390 b | 1350 b | 14.2 b | 29.5 b | 91.8 a | 160.0 b | 13 b | 27 b | |
| **Biostimulant (B)** |  |  |  |  |  |  |  |  | |
| **Control** | 200 a | 770 a | 11.8 | 19.0 a | 92.6 a | 117.0 a | 9.1 a | 21 a | |
| **ANE A** | 330 b | 950 b | 11.7 | 20.5 b | 104.0 b | 127.0 b | 12 b | 23 b | |
| **N X B** |  |  |  |  |  |  |  |  | |
| **0 MS x Control** | 100 a | 310 | 11.3 b | 9.2 | 101.0 | 82.9 | 7.2 a | 16 | |
| **0 MS x ANE A** | 170 b | 440 | 7.1 a | 10.8 | 108.0 | 86.4 | 8.7 a | 17 | |
| **1/10 MS x Control** | 310 c | 1220 | 12.2 b | 28.7 | 84.0 | 152.0 | 11 b | 26 | |
| **1/10 MS x ANE A** | 480 d | 1470 | 16.3 c | 30.2 | 99.5 | 168.0 | 15 c | 29 | |
| **Statistical significance** | | | | | | | | |  |
| **Nutrient (N)** | ******* | ******* | ******* | ******* | ******* | ******* | ******* | ******* | |
| **Biostimulant (B)** | ******* | ******* | **ns** | ***** | ***** | ***** | ******* | ****** | |
| **N × B** | ******* | **ns** | ******* | **ns** | **ns** | **ns** | ****** | **ns** | |

All data are expressed as average per sample in 12-day old plant seedlings. ns, *, **, and *** means non-significant or significant at *p* ≤ 0.05, *p* ≤ 0.01, and *p* ≤ 0.001, respectively. Different letters indicate statistical differences with *p* ≤ 0.05 based on t-test for N and B factors and one-way ANOVA for N x B factor. Number of biological replicates (n ≥ 4).

Table S6. Effect of nutrient conditions and root application of ANE A on macro- and secondary nutrient content in wheat tissues

| **Source of variance** | **Content K (µg)** | | **Content Ca (µg)** | | **Content P (µg)** | | **Content Mg (µg)** | | |
| --- | --- | --- | --- | --- | --- | --- | --- | --- | --- |
|  | **Root** | **Shoot** | **Root** | **Shoot** | **Root** | **Shoot** | **Root** | **Shoot** | |
| **Nutrient (N)** |  |  |  |  |  |  |  |  | |
| **0 MS** | 69 a | 357 a | 5.7 a | 11.3 a | 63 a | 130 a | 9.5 a | 36.1 a | |
| **1/10 MS** | 304 b | 843 b | 10.9 b | 27.9 b | 81 b | 190 b | 16.5 b | 44.1 b | |
| **Biostimulant (B)** |  |  |  |  |  |  |  |  | |
| **Control** | 157 a | 555 a | 8.1 | 19.1 | 64 a | 150 | 12.2 | 38.1 a | |
| **ANE A** | 216 b | 645 b | 8.5 | 20.2 | 80 b | 170 | 13.7 | 42.1 b | |
| **N X B** |  |  |  |  |  |  |  |  | |
| **0 MS x Control** | 57 a | 297 | 5.6 | 10.9 | 56 | 120 | 8.8 | 34.7 | |
| **0 MS x ANE A** | 80 a | 417 | 5.8 | 11.7 | 70 | 140 | 10.1 | 37.5 | |
| **1/10 MS x Control** | 256 b | 696 | 10.5 | 27.2 | 72 | 180 | 15.7 | 41.4 | |
| **1/10 MS x ANE A** | 351 c | 874 | 11.2 | 28.7 | 90 | 190 | 17.4 | 46.7 | |
| **Statistical significance** | | | | | | | | |  |
| **Nutrient (N)** | ******* | ******* | ******* | ******* | ******* | ******* | ******* | ******* | |
| **Biostimulant (B)** | ******* | ***** | **ns** | **ns** | ******* | **ns** | **ns** | ***** | |
| **N × B** | ******* | **ns** | **ns** | **ns** | **ns** | **ns** | **ns** | **ns** | |

All data are expressed as average per sample in 12-day old plant seedlings. ns, *, **, and *** means non-significant or significant at *p* ≤ 0.05, *p* ≤ 0.01, and *p* ≤ 0.001, respectively. Different letters indicate statistical differences with *p* ≤ 0.05 based on t-test for N and B factors and one-way ANOVA for N x B factor. Number of biological replicates (n ≥ 4).

Table S7. Effect of nutrient conditions and foliar application of ANE A on micronutrient content in barley tissues

| **Source of variance** | **Content B (µg)** | | **Content Mn (µg)** | | **Content Cu (µg)** | | **Content Fe (µg)** | | **Content Mo (ng)** | | **Content Zn (µg)** | |
| --- | --- | --- | --- | --- | --- | --- | --- | --- | --- | --- | --- | --- |
|  | **Root** | **Shoot** | **Root** | **Shoot** | **Root** | **Shoot** | **Root** | **Shoot** | **Root** | **Shoot** | **Root** | **Shoot** |
| **Nutrient (N)** |  |  |  |  |  |  |  |  |  |  |  |  |
| **0 MS** | 0.11 a | 0.20 a | 0.27 a | 0.82 a | 0.17 b | 0.32 a | 1.0 a | 2.4 a | 0.6 a | 7.8 a | 0.69 a | 1.25 a |
| **1/10 MS** | 0.15 b | 0.23 b | 3.93 b | 2.97 b | 0.15 a | 0.44 b | 3.3 b | 3.7 b | 51.1 b | 153.0 b | 0.93 b | 1.97 b |
| **Biostimulant (B)** |  |  |  |  |  |  |  |  |  |  |  |  |
| **Control** | 0.12 a | 0.20 a | 1.95 a | 1.91 | 0.15 a | 0.38 | 2.3 a | 2.7 a | 21.6 a | 71.9 a | 0.76 a | 1.52 a |
| **ANE A** | 0.14 b | 0.23 b | 2.26 b | 1.87 | 0.17 b | 0.38 | 2.7 b | 3.4 b | 30.1 b | 88.8 b | 0.86 b | 1.69 b |
| **N X B** |  |  |  |  |  |  |  |  |  |  |  |  |
| **0 MS x Control** | 0.10 | 0.16 a | 0.25 a | 0.79 a | 0.14 a | 0.29 a | 1.2 a | 2.1 | 0.0 a | 5.8 a | 0.64 | 1.13 |
| **0 MS x ANE A** | 0.11 | 0.23 b | 0.29 a | 0.85 a | 0.20 b | 0.34 b | 2.0 b | 2.8 | 1.1 a | 9.9 a | 0.75 | 1.37 |
| **1/10 MS x Control** | 0.14 | 0.23 b | 3.62 b | 3.04 b | 0.16 a | 0.46 d | 3.4 c | 3.4 | 43.3 b | 138.0 b | 0.88 | 1.91 |
| **1/10 MS x ANE A** | 0.16 | 0.23 b | 4.23 c | 2.89 b | 0.14 a | 0.42 c | 3.3 c | 4.1 | 59.0 c | 168.0 c | 0.98 | 2.02 |
| **Statistical significance** | | | | | | | | | | | | |
| **Nutrient (N)** | ******* | ******* | ******* | ******* | ****** | ******* | ******* | ******* | ******* | ******* | ******* | ******* |
| **Biostimulant (B)** | ******* | ******* | ******* | **ns** | ***** | **ns** | ******* | ******* | ******* | ******* | ****** | ******* |
| **N × B** | **ns** | ******* | ****** | ***** | ******* | ******* | ******* | **ns** | ******* | ******* | **ns** | **ns** |

All data are expressed as average per sample in 12-day old plant seedlings. ns, *, **, and *** means non-significant or significant at *p* ≤ 0.05, *p* ≤ 0.01, and *p* ≤ 0.001, respectively. Different letters indicate statistical differences with *p* ≤ 0.05 based on t-test for N and B factors and one-way ANOVA for N x B factor. Number of biological replicates (n ≥ 3).

Table S8. Effect of nutrient conditions and root application of ANE A on micronutrient content in barley tissues

| **Source of variance** | **Content B (µg)** | | **Content Mn (µg)** | | **Content Cu (µg)** | | **Content Fe (µg)** | | **Content Mo (ng)** | | **Content Zn (µg)** | |
| --- | --- | --- | --- | --- | --- | --- | --- | --- | --- | --- | --- | --- |
|  | **Root** | **Shoot** | **Root** | **Shoot** | **Root** | **Shoot** | **Root** | **Shoot** | **Root** | **Shoot** | **Root** | **Shoot** |
| **Nutrient (N)** |  |  |  |  |  |  |  |  |  |  |  |  |
| **0 MS** | 0.11 | 0.20 | 0.30 a | 0.42 a | 0.12 | 0.16 a | 1.1 a | 1.6 | 8.60 a | 20.0 a | 0.57 | 0.73 a |
| **1/10 MS** | 0.12 | 0.21 | 1.87 b | 1.01 b | 0.12 | 0.20 b | 2.1 b | 1.8 | 23.0 b | 38.0 b | 0.61 | 0.87 b |
| **Biostimulant (B)** |  |  |  |  |  |  |  |  |  |  |  |  |
| **Control** | 0.11 | 0.21 | 1.05 | 0.69 a | 0.11 a | 0.18 | 1.5 | 1.6 | 15.0 a | 27.0 a | 0.61 | 0.79 |
| **ANE A** | 0.12 | 0.20 | 1.11 | 0.74 b | 0.13 b | 0.18 | 1.6 | 1.7 | 17.0 b | 33.0 b | 0.58 | 0.80 |
| **N X B** |  |  |  |  |  |  |  |  |  |  |  |  |
| **0 MS x Control** | 0.11 | 0.22 ab | 0.29 | 0.41 | 0.12 | 0.16 | 0.9 | 1.6 | 7.60 | 19.0 | 0.56 a | 0.74 a |
| **0 MS x ANE A** | 0.11 | 0.17 a | 0.31 | 0.42 | 0.13 | 0.16 | 1.2 | 1.6 | 9.70 | 22.0 | 0.58 ab | 0.71 a |
| **1/10 MS x Control** | 0.11 | 0.20 ab | 1.82 | 0.98 | 0.11 | 0.20 | 2.1 | 1.6 | 23.0 | 36.0 | 0.65 b | 0.84 b |
| **1/10 MS x ANE A** | 0.12 | 0.22 b | 1.91 | 1.05 | 0.14 | 0.19 | 2.1 | 1.9 | 24.0 | 41.0 | 0.57 a | 0.89 b |
| Statistical significance | | | | | | | | | | | | |
| **Nutrient (N)** | **ns** | **ns** | ******* | ******* | **ns** | ****** | ******* | **ns** | ******* | ******* | **ns** | ******* |
| **Biostimulant (B)** | **ns** | **ns** | **ns** | ***** | ***** | **ns** | **ns** | **ns** | ***** | ******* | **ns** | **ns** |
| **N × B** | **ns** | ***** | **ns** | **ns** | **ns** | **ns** | **ns** | **ns** | **ns** | **ns** | ***** | ***** |

All data are expressed as average per sample in 12-day old plant seedlings. ns, *, **, and *** means non-significant or significant at *p* ≤ 0.05, *p* ≤ 0.01, and *p* ≤ 0.001, respectively. Different letters indicate statistical differences with *p* ≤ 0.05 based on t-test for N and B factors and one-way ANOVA for N x B factor. Number of biological replicates (n ≥ 3).

Table S9. Effect of nutrient conditions and foliar application of ANE A on micronutrient content in wheat tissues

| **Source of variance** | **Content B (µg)** | | **Content Mn (µg)** | | **Content Cu (µg)** | | **Content Fe (µg)** | | **Content Mo (ng)** | | **Content Zn (µg)** | |
| --- | --- | --- | --- | --- | --- | --- | --- | --- | --- | --- | --- | --- |
|  | **Root** | **Shoot** | **Root** | **Shoot** | **Root** | **Shoot** | **Root** | **Shoot** | **Root** | **Shoot** | **Root** | **Shoot** |
| **Nutrient (N)** |  |  |  |  |  |  |  |  |  |  |  |  |
| **0 MS** | 0.47 a | 0.11 a | 0.18 a | 0.36 a | 55 | 68 a | 1.4 a | 1.4 a | 0 a | 2.8 a | 0.49 a | 0.37 a |
| **1/10 MS** | 0.19 b | 0.15 b | 1.80 b | 1.82 b | 59 | 120 b | 2.0 b | 2.4 b | 9.6 b | 59 b | 0.55 b | 0.98 b |
| **Biostimulant (B)** |  |  |  |  |  |  |  |  |  |  |  |  |
| **Control** | 0.40 b | 0.13 b | 0.84 a | 1.06 | 50 a | 84 a | 1.8 b | 1.8 a | 4.1 a | 27 a | 0.48 a | 0.62 a |
| **ANE A** | 0.26 a | 0.12 a | 1.13 b | 1.12 | 64 b | 100 b | 1.6 a | 2.1 b | 5.5 b | 35 b | 0.57 b | 0.72 b |
| **N X B** |  |  |  |  |  |  |  |  |  |  |  |  |
| **0 MS x Control** | 0.62 c | 0.12 b | 0.16 a | 0.34 | 49 | 69 a | 1.5 | 1.3 | 0 a | 2.1 a | 0.44 | 0.33 |
| **0 MS x ANE A** | 0.31 b | 0.10 a | 0.19 a | 0.38 | 61 | 67 a | 1.4 | 1.5 | 0 a | 3.5 a | 0.55 | 0.41 |
| **1/10 MS x Control** | 0.18 a | 0.15 c | 1.52 b | 1.78 | 50 | 99 b | 2.1 | 2.2 | 8.1 b | 51 b | 0.52 | 0.92 |
| **1/10 MS x ANE A** | 0.20 a | 0.15 c | 2.07 c | 1.86 | 67 | 130 c | 1.8 | 2.6 | 11 c | 66 c | 0.59 | 1.00 |
| **Statistical significance** | | | | | | | | | | | | |
| **Nutrient (N)** | ******* | ******* | ******* | ******* | **ns** | ******* | ******* | ******* | ******* | ******* | ***** | ******* |
| **Biostimulant (B)** | ******* | ****** | ******* | **ns** | ******* | ******* | ****** | ******* | ******* | ******* | ****** | ******* |
| **N × B** | ******* | ****** | ******* | **ns** | **ns** | ******* | **ns** | **ns** | ******* | ******* | **ns** | **ns** |

All data are expressed as average per sample in 12-day old plant seedlings. ns, *, **, and *** means non-significant or significant at *p* ≤ 0.05, *p* ≤ 0.01, and *p* ≤ 0.001, respectively. Different letters indicate statistical differences with *p* ≤ 0.05 based on t-test for N and B factors and one-way ANOVA for N x B factor. Number of biological replicates (n ≥ 3).

Table S10. Effect of nutrient conditions and root application of ANE A on micronutrient content in wheat tissues

| **Source of variance** | **Content B (µg)** | | **Content Mn (µg)** | | **Content Cu (µg)** | | **Content Fe (µg)** | | **Content Mo (ng)** | | **Content Zn (µg)** | |
| --- | --- | --- | --- | --- | --- | --- | --- | --- | --- | --- | --- | --- |
|  | **Root** | **Shoot** | **Root** | **Shoot** | **Root** | **Shoot** | **Root** | **Shoot** | **Root** | **Shoot** | **Root** | **Shoot** |
| **Nutrient (N)** |  |  |  |  |  |  |  |  |  |  |  |  |
| **0 MS** | 0.07 a | 0.16 a | 0.21 a | 0.66 a | 0.10 a | 0.12 a | 0.53 a | 1.30 a | 1.70 a | 2.4 a | 0.32 a | 0.58 a |
| **1/10 MS** | 0.10 b | 0.19 b | 1.40 b | 1.70 b | 0.34 b | 0.25 b | 0.80 b | 3.44 b | 8.42 b | 27 b | 0.58 b | 0.92 b |
| **Biostimulant (B)** |  |  |  |  |  |  |  |  |  |  |  |  |
| **Control** | 0.08 | 0.18 | 0.70 a | 1.15 | 0.19 a | 0.17 | 0.65 | 1.33 a | 4.41 a | 11 a | 0.44 | 0.75 |
| **ANE A** | 0.09 | 0.17 | 0.90 b | 1.21 | 0.25 b | 0.19 | 0.69 | 3.41 b | 5.72 b | 18 b | 0.46 | 0.75 |
| **N X B** |  |  |  |  |  |  |  |  |  |  |  |  |
| **0 MS x Control** | 0.07 | 0.17 | 0.19 a | 0.65 | 0.09 a | 0.11 | 0.53 | 1.20 a | 1.40 a | 2.7 a | 0.32 | 0.57 |
| **0 MS x ANE A** | 0.08 | 0.15 | 0.22 a | 0.66 | 0.11 a | 0.12 | 0.53 | 1.39 a | 2.01 a | 2.1 a | 0.32 | 0.58 |
| **1/10 MS x Control** | 0.09 | 0.19 | 1.21 b | 1.65 | 0.29 b | 0.23 | 0.76 | 1.45 a | 7.42 b | 20 b | 0.56 | 0.92 |
| **1/10 MS x ANE A** | 0.10 | 0.19 | 1.58 c | 1.75 | 0.40 c | 0.26 | 0.85 | 5.43 b | 9.42 c | 34 c | 0.60 | 0.91 |
| **Statistical significance** | | | | | | | | | | | | |
| **Nutrient (N)** | ******* | ****** | ******* | ******* | ******* | ******* | ******* | ****** | ******* | ******* | ******* | ******* |
| **Biostimulant (B)** | **ns** | **ns** | ******* | **ns** | ******* | **ns** | **ns** | ****** | ******* | ******* | **ns** | **ns** |
| **N × B** | **ns** | **ns** | ****** | **ns** | ******* | **ns** | **ns** | ****** | ****** | ******* | **ns** | **ns** |

All data are expressed as average per sample in 12-day old plant seedlings. ns, *, **, and *** means non-significant or significant at *p* ≤ 0.05, *p* ≤ 0.01, and *p* ≤ 0.001, respectively. Different letters indicate statistical differences with *p* ≤ 0.05 based on t-test for N and B factors and one-way ANOVA for N x B factor. Number of biological replicates (n ≥ 3).
